# Discovery of potent anti-inflammatory flavonoid-sesquiterpenoid hybrids from *Syzygium simile*: Structure–activity relationships and nanomolar inhibition of human neutrophil activation

**DOI:** 10.1080/13880209.2026.2746239

**Published:** 2026-10-28

**Authors:** Yu-Chun Lin, Ho-Cheng Wu, Tsong-Long Hwang, Yueh-Hsiung Kuo, Chu-Hung Lin, Horng-Huey Ko, Hsun-Shuo Chang

**Affiliations:** ^a^ School of Pharmacy, Kaohsiung Medical University, Kaohsiung, Taiwan; ^b^ Department of Medical Research, Kaohsiung Medical University Hospital, Kaohsiung, Taiwan; ^c^ Graduate Institute of Health Industry Technology and Center for Drug Research and Development, Chang Gung University of Science and Technology, Taoyuan, Taiwan; ^d^ Department of Chinese Pharmaceutical Sciences and Chinese Medicine Resources, Chinese Medicine Research Center, China Medical University, Taichung, Taiwan; ^e^ Department of Biotechnology, Asia University, Taichung, Taiwan; ^f^ Biomedical Technology and Device Research Laboratories, Industrial Technology Research Institute, Hsinchu, Taiwan; ^g^ Drug Development and Value Creation Research Center, Kaohsiung Medical University, Kaohsiung, Taiwan; ^h^ Department of Pharmacy, Kaohsiung Municipal Siaogang Hospital, Kaohsiung, Taiwan

**Keywords:** Myrtaceae, *Syzygium simile*, flavonoid-sesquiterpenoid hybrids, anti-inflammatory activity

## Abstract

**Context:**

Current anti-inflammatory drugs often cause significant adverse effects, underscoring the need for safer alternatives. Natural products with rare and distinctive scaffolds represent a promising source of leads for discovering new anti-inflammatory agents.

**Objective:**

This study aimed to find anti-inflammatory flavonoid-sesquiterpenoid hybrids from *Syzygium simile* (Myrtaceae), and to clarify the structural-activity relationship underlying their inhibition of biomarkers in activated human neutrophils.

**Materials and methods:**

A range of chromatographic techniques was sequentially applied to isolate pure compounds, the structures of which were elucidated by spectroscopic and spectrometric analyses. Anti-inflammatory activities were evaluated in fMLP/CB-induced human neutrophils by measuring inhibitory effects on superoxide anion generation and elastase release.

**Results:**

Twenty-two flavonoid-sesquiterpenoid hybrids, including thirteen previously undescribed structures, were isolated from *S. simile* leaves, comprising five flavonoid scaffolds (types I–V) and five sesquiterpenoid moieties (germacrene-, cubebene-, oplopene-, cadinene-, and copaene-types) generated via distinctive electron transfer-mediated biosynthesis. Most compounds potently inhibited superoxide anion generation and elastase release, with IC_50_ values up to 32-fold lower than that of the positive control, without exhibiting cytotoxicity.

**Discussion and Conclusion:**

Structure-activity relationship analysis revealed that the combination of a type I flavonoid and a germacrene-type sesquiterpenoid skeleton, integrated through an rA-type ring fusion, represents the optimal pharmacophore. The lead compound, simisyzygin C (**3**), achieved nanomolar potency with IC_50_ values of 0.04 μM (superoxide anion) and 0.16 μM (elastase release). These findings identify flavonoid-sesquiterpenoid hybrids as a structurally distinctive class of anti-inflammatory natural products, providing a structural basis for lead optimization.

## Introduction

Inflammation represents a complex protective response of the body to infections, injuries, or irritants, aimed at eliminating the primary stimulus, clearing damaged cells, and initiating tissue repair. This process is characterized by five cardinal signs: redness, swelling, heat, pain, and loss of function. However, excessive inflammatory responses can lead to massive cytokine release, causing tissue damage and potentially resulting in organ failure in severe cases (Chen et al. 2018). When inflammation fails to resolve, the condition evolves into chronic inflammation, leading to the development of various chronic inflammatory diseases such as type 2 diabetes, cardiovascular disease, arthritis, Alzheimer’s disease, chronic kidney disease, and cancer (Furman et al. 2019).

Current clinical anti-inflammatory medications present significant limitations, including restricted therapeutic efficacy and various side effects or adverse reactions. Non-steroidal anti-inflammatory drugs (NSAIDs) inhibit cyclooxygenase but are associated with gastrointestinal ulceration, renal impairment, elevated cardiovascular risk, and allergic reactions (Pirmohamed et al. 2004; Howard et al. 2007). Corticosteroids suppress phospholipase A2; however, their prolonged use may lead to osteoporosis, fat redistribution, Cushing’s syndrome, and increased susceptibility to infection (Schäcke et al. 2002). As H1 and H2 receptor antagonists, antihistamines frequently cause central nervous system side effects, including sedation, drowsiness, and anticholinergic effects (Li et al. 2022). Similarly, immunomodulators and biologics may increase infection risk, precipitate allergic reactions, and, in rare cases, elevate malignancy risk (Bonovas et al. 2016; Rubbert-Roth 2012). These therapeutic challenges highlight the urgent need to discover novel anti-inflammatory lead compounds.

Neutrophils, as the most abundant and key circulating leukocytes in the innate immune system, constitute the first line of defense, primarily functioning through the massive production of superoxide anions and related reactive oxygen species (ROS), proteolytic enzymes, neutrophil extracellular traps (NETs), and cytokines (Chen et al. 2025; Nauseef et al. 2014). However, dysregulated neutrophils can trigger or promote various inflammatory responses, causing damage to surrounding tissues, including acute respiratory distress syndrome (ARDS), coronavirus disease (COVID-19)-associated ARDS, chronic obstructive pulmonary disease (COPD), and psoriasis (Soehnlein et al. 2017; Chiang et al. 2019; 2020; Yang et al. 2021). Therefore, targeted modulation of neutrophil activity provides an effective therapeutic strategy for alleviating excessive inflammation.

The Myrtaceae family represents the eighth largest flowering plant family worldwide, comprising approximately 132 genera and nearly 6,000 species distributed across tropical and subtropical regions globally (Christenhusz et al. 2016). Within this family, the genus *Syzygium* stands as one of the most important genera. The genus name “*Syzygium*” derives from Greek, meaning “coupled,” reflecting its distinctive paired leaves and branches as morphological characteristics (Nigam et al. 2012). This genus encompasses 1,200-1,800 species with an extensive distribution range spanning from Africa and Madagascar through Asia to the entire Pacific region (Tuiwawa et al. 2013; Ahmad et al. 2016). The genus possesses diverse pharmacological activities, including antibacterial, antiviral, anticancer, antithrombotic, hypoglycemic, and anti-inflammatory effects, while demonstrating remarkable chemical structural diversity encompassing flavonoids, triterpenes, tannins, and phloroglucinol derivatives (Cock et al. 2018; Batiha et al. 2020; Uddin et al. 2022; Saber et al. 2024).

*Syzygium simile* (Merr.) Merr., commonly known as “Lanyu-chinan,” represents one of Taiwan’s twelve native *Syzygium* species. This species is indigenous to the Philippines and Taiwan, particularly on Lanyu (Orchid Island) and Lyudao (Green Island) (Chang et al. 1993). From an ethnobotanical perspective, the Yami indigenous people utilize the timber of *S. simile* for constructing traditional plank boats, and its fruits are called “pangoon” in the Yami language, meaning “edible” (Yan 2012). However, no reports exist regarding its traditional medicinal uses.

The phytochemical composition and bioactive potential of *S. simile* remained largely unexplored in scientific literature until recently. In our previous investigation targeting metabolic-associated fatty liver disease (MAFLD) activity, we discovered that the methanol extract of *S. simile* leaves significantly inhibited lipid droplet accumulation by 40% at 25 μM (Yen et al. 2018). From this extract, we identified nine structurally unique compounds classified as flavonoid-sesquiterpenoid hybrids, designated as simisyzygins C-G (**3**–**7**) and simicadinene A-D (**19**–**22**) (Figure 1) (Yang et al. 2025). During subsequent reexamination, two compounds previously reported as known structures were re-determined to be new stereoisomers, simisyzygins A-B (**1**–**2**), based on X-ray crystallographic and circular dichroism (CD) analyses.

**Figure 1.**
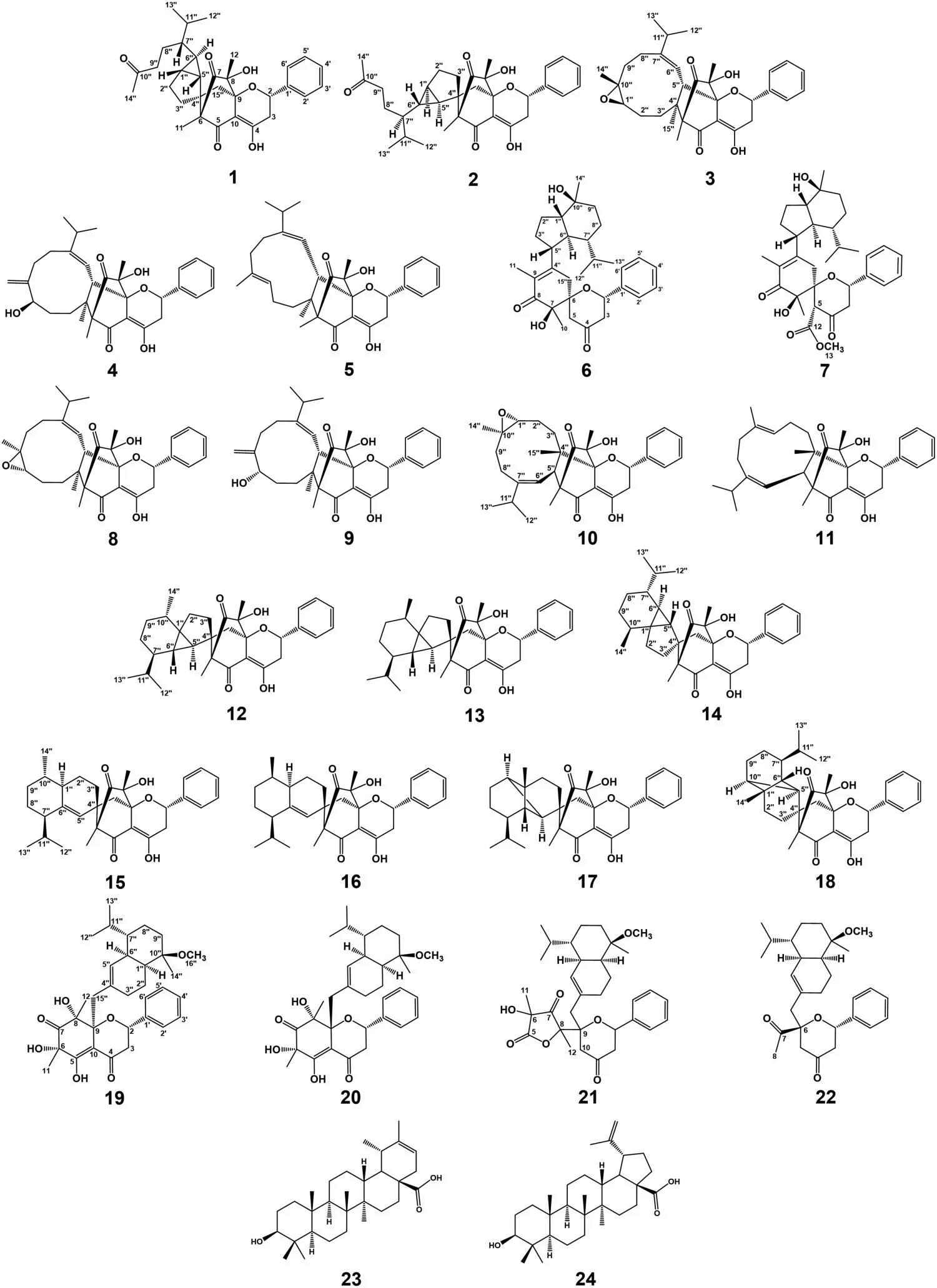
Structure of compounds **1**–**24**.

These novel hybrids exhibit unique structural characteristics and diverse molecular configurations unprecedented in nature, demonstrating remarkable structural diversity and promising biological activities. Therefore, exploring their anti-inflammatory potential represents a highly valuable research direction. Thus, in further exploration of *S. simile*, we successfully isolated eleven additional novel hybrids, simisyzygins H1-H4 (**8**–**11**), I1-I3 (**12**–**14**), J1-J2 (**15**–**16**), and K1-K2 (**17**–**18**), along with two known triterpenoids, 3*β*-hydroxytaraxast-20-en-28-oic acid (**23**) and betulinic acid (**24**) (Figure 1). Collectively, the complete series of 22 hybrids comprised five types of flavonoid scaffolds (I–V) and five types of sesquiterpenoid moieties, including germacrene-type (**3**–**5**, and **8**–**11**), cubebene-type (**1**–**2**, and **12**–**14**), oplopene-type (**6**–**7**), cadinene-type (**15**–**16**, and **19**–**22**), and copaene-type (**17**–**18**). These structural units were integrated through various connection modes, such as ring fusion, spiro connection, or single bond connection, forming a structurally diverse compound library. The results of inhibition assays of human neutrophil-mediated superoxide anion generation and elastase release demonstrated that these hybrids could offer more diverse and safer therapeutic options to overcome the limitations of existing anti-inflammatory medications.

## Materials and methods

### General experimental procedures

Optical rotation measurements were performed on a Jasco P-2000 polarimeter. UV spectra were obtained with a Hitachi U-5100 UV/VIS spectrophotometer. CD spectra were measured on a Jasco J-815 CD spectrometer. IR spectra were recorded in ATR mode on a Jasco FT/IR-4600 spectrometer. The 1D (^1^H,^13^C, DEPT) and 2D (COSY, NOESY, HSQC, HMBC) NMR spectra, detected using CDCl_3_ solvent, were recorded on a Varian Germini-2000 spectrometer operated at 200 (^1^H) and 50 MHz (^13^C), a Varian Unityplus-400 spectrometer operated at 400 (^1^H) and 100 MHz (^13^C), a Varian Mercuryplus-400 spectrometer operated at 400 (^1^H) and 100 MHz (^13^C), and a Varian VNMRS-600 spectrometer operated at 600 (^1^H) and 150 MHz (^13^C). Chemical shifts (*δ*) were referenced to residual CDCl_3_ solvent signals as an internal standard (^1^H: *δ* 7.26; ^13^C: *δ* 77.0). Low-resolution mass spectra were recorded on a Thermo Fisher Orbitrap Exploris 120 (ESIMS). High-resolution mass spectra were recorded on a Bruker Solarix FT-MS (ESIMS). Silica gel (CHROMATOREX SMB100-20/45) and reversed phase silica gel (YMC*GEL^®^ ODS-A-HG 12 nm S-50 µm) were used for column chromatography, and Merck Art. 105554 TLC Silica gel 60 F254 and Merck Art. 105559 TLC Silica gel 60 RP-18 F254S were used for TLC and preparative TLC; spots were visualized with a Ce_2_(SO_4_)_3_ aqueous solution.

Purification was performed by (i) Medium-Performance Liquid Chromatography (MPLC) using an EYELA VSP-2050 ceramic pump, (ii) flash chromatography on a PuriFlash^®^ XS420 system, and (iii) High-Performance Liquid Chromatography (HPLC) using Phenomenex 250 × 10.0 mm, Purospher^®^ STAR Phenyl (5 μm) Hibar^®^ RT 250-10, and YMC-Pack SIL SIL-06 columns, the system being equipped with a HITACHI L-7100 pump system and a JASCO UV-975 detector.

### Plant material

The leaves of *S. simile* were collected from Mudan Township, Pingtung County, Taiwan, in August 2017, and were botanically identified by Prof. Ih-Sheng Chen (Kaohsiung Medical University, Taiwan). A voucher specimen (no. Chen 5621) has been deposited in the Herbarium of the School of Pharmacy, College of Pharmacy, Kaohsiung Medical University.

### Extraction and isolation

Dried leaves of *S. simile* (5.5 kg) were extracted with cold MeOH (60 L) at 25 °C three times, three days each time, to yield a MeOH extract (650 g). The MeOH extract was partitioned between an *n*-hexane layer (220 g) and an H_2_O layer (22.5 L). The H_2_O layer was subsequently partitioned between an EtOAc layer (100 g) and an H_2_O layer (240 g). All fractions were evaluated for anti-inflammatory activity, with both the *n*-hexane and EtOAc layers showing promising results. The bioactive *n*-hexane layer was fractionated using silica gel flash column chromatography (CC) with a gradient of *n*-hexane/acetone (98:2 to 50:50, v/v), followed by 100% acetone, and 100% MeOH to give fourteen fractions (Frs. 1 ∼ 14). Compounds **3**–**7** and **19**–**22** were isolated following the procedure described previously (Yang et al. 2025). Fr. 7 (5.8 g) was subjected to silica gel flash column chromatography, eluted with a gradient of *n*-hexane/EtOAc (88:12, v/v) to (75:25, v/v), 100% EtOAc, and 100% MeOH, furnishing 13 fractions (Frs. 7-1 ∼ 7–13). The combination of Frs. 7–9 (595.0 mg) and 7–10 (311.4 mg) was separated through C18 reversed-phase chromatography with a gradient of H_2_O/MeOH (1:8, v/v) to obtain 14 fractions (Frs. 7-9-1 ∼ 7-9-14). Frs. 7-9-2 (80.0 mg) and 7-9-3 (41.8 mg) were pooled and purified on silica gel MPLC, under CH_2_Cl_2_/EtOAc (9:1, v/v) to give 8 fractions (Frs. 7-9-2-1 ∼ 7-9-2-8). Subsequent purification of Fr. 7-9-2-5 (38.7 mg) on silica gel chromatography with *n*-hexane/EtOAc (7:2, v/v) led to **9** (1.0 mg). Fr. 7-9-7 (593.8 mg) was processed through silica gel MPLC, with *n*-hexane/EtOAc (1:5, v/v) to yield 7 fractions (Frs. 7-9-7-1 ∼ 7-9-7-7). Further fractionation of Fr. 7-9-7-2 (265.8 mg) on CHROMATOREX SMB100-20/45 using *n*-hexane/EtOAc (3:1, v/v) resulted in **8** (4.9 mg), **14** (1.5 mg), and **18** (0.8 mg). Separation of Fr. 7-9-7-4 (185.8 mg) through C18 reversed-phase MPLC, under H_2_O/acetone (1:3, v/v), gave 5 fractions (Frs. 7-9-7-4-1 ∼ 7-9-7-4-5). Subsequent processing of Fr. 7-9-7-4-1 (18.5 mg) through MPLC with *n*-hexane/EtOAc (4:1, v/v) obtained **10** (0.6 mg) and **11** (3.2 mg). Fr. 7-9-7-4-3 (162.5 mg) was subjected to silica gel MPLC, eluted with *n*-hexane/EtOAc (5:1, v/v), yielding 9 fractions (Frs. 7-9-7-4-3-1 ∼ 7-9-7-4-3-9) and affording **12** (163.5 mg). Fr. 7-9-7-4-3-6 (8.0 mg) was separated by reversed-phase YMC gel^®^ C18 with H_2_O/MeOH (5:1, v/v) to obtain **13** (0.8 mg). Refinement of Fr. 7-9-7-4-3-7 (2.2 mg) via silica gel HPLC (Phenomenex 250 × 10.0 mm) employing *n*-hexane/EtOAc (3:1, v/v) furnished **17** (1.7 mg). Chromatographic separation of Fr. 7-9-8 (61.0 mg) on reversed-phase YMC gel^®^ C18 using H_2_O/MeOH (1:10, v/v) afforded 8 fractions (Frs. 7-9-8-1 ∼ 7-9-8-8). Further processing of Fr. 7-9-8-7 (14.5 mg) through silica gel MPLC with a gradient of *n*-hexane/CH_2_Cl_2_/EtOAc (8:1:1, v/v/v), afforded **15** (1.6 mg) and **16** (0.4 mg), respectively. Fraction 10 (10.0 g) was fractionated by silica gel flash chromatography using a gradient of CH_2_Cl_2_/MeOH (100:0 to 50:1, v/v), followed by pure acetone and MeOH, yielding 14 subfractions (Frs. 10-1 to 10–14). Fr. 10-6 was further separated on reversed-phase C_18_ silica gel with H_2_O/MeOH (1:3 to 1:5, v/v), then 100% MeOH and 100% acetone, affording ten subfractions (Frs. 10-6-1 to 10-6-10). Subsequent purification of Fr. 10-6-7 by silica gel MPLC using CH_2_Cl_2_/EtOAc and CH_2_Cl_2_/acetone gradients, followed by MPLC with H_2_O/acetonitrile (1:3, v/v), yielded **1** (9.2 mg) and **2** (21.8 mg). All compounds were determined to be >95% pure by HPLC.

### Simisyzygin A (1)

Colorless prisms; ${\left[\alpha \right]}_{D}^{21}$ −137.1 (*c* 0.46, MeOH); UV (MeOH) *λ*_max_ (log *ε*) 240 (3.51), 288 (3.98) nm; CD (MeOH) *λ*_ext_ (Δ*ε*) 236 (−2.55), 291 (+23.34), 335 (−21.76) nm; IR (ATR) *ν*_max_ 3401 (OH), 1724, 1711, 1659 (C = O), 1610 (aromatic ring) cm^−1^; ^1^H NMR and ^13^C NMR data, Table 1; ESIMS *m/z* 521 [M + H]^+^; HRESIMS *m/z* 543.27286 [M + Na]^+^ (calcd for C_32_H_40_NaO_6_, 543.27226).

**Table 1.** ^1^H and ^13^C NMR Data for **1**–**2** in CDCl_3_.

	1^a^		2^a^	
Position	*δ*_H_ (*J* in Hz)	*δ*_C_, type	*δ*_H_ (*J* in Hz)	*δ*_C_, type
2	5.77, dd (9.2, 3.6)	74.0, CH	5.17, dd (10.0, 3.6)	74.1, CH
3a	2.70, dd (17.8, 9.2)	35.7, CH_2_	2.72, dd (17.8, 10.0)	35.9, CH_2_
3b	2.62, dd (17.8, 3.6)		2.63, dd (17.8, 3.6)	
4		169.7, C		169.1, C
5		195.5, C		195.6, C
6		68.7, C		66.1, C
7		208.4, C		208.3, C
8		77.2, C		77.4, C
9		76.4, C		76.5, C
10		109.0, C		108.9, C
11	1.16, s	7.6, CH_3_	1.26, s	6.8, CH_3_
12	1.46, s	22.5, CH_3_	1.44, s	22.6, CH_3_
1′		140.1, C		140.2, C
2′, 6′	7.39, m	126.4, CH	7.39, m	126.2, CH
3′, 5′	7.39, m	128.7, CH	7.39, m	128.7, CH
4′	7.36, m	128.5, CH	7.35, m	128.4, CH
1ʺ	1.15, m	28.0, CH	0.99, m	22.1, CH
2ʺa	1.69, m	29.2, CH_2_	1.71, m	25.4, CH_2_
2ʺb	1.69, m		1.71, m	
3ʺa	1.74, m	38.1, CH_2_	1.19, m	30.5, CH_2_
3ʺb	1.07, m		1.16, m	
4ʺ		49.7, C		49.2, C
5ʺ	0.91, dd (6.0, 3.2)	32.6, CH	0.63, t (6.3)	33.7, CH
6ʺ	0.34, dt (10.0, 3.2)	23.7, CH	0.62, dt (12.0, 3.6)	24.9, CH
7ʺ	0.45, m	47.0, CH	0.43, m	42.7, CH
8ʺa	1.63, m	24.1, CH_2_	1.63, m	23.6, CH_2_
8ʺb	1.48, m		1.44, m	
9ʺa	2.53, ddd (16.0, 10.0, 5.6)	42.5, CH_2_	2.53, ddd (16.4, 10.0, 4.0)	42.4, CH_2_
9ʺb	2.38, ddd (16.0, 9.6, 5.6)		2.39, ddd (16.4, 9.6, 6.0)	
10ʺ		209.4, C		209.3, C
11ʺ	1.76, septd (7.0, 2.8)	30.9, CH	1.79, septd (6.8, 2.8)	30.6, CH
12ʺ	0.87, d (7.0)	18.0, CH_3_	0.92, d (6.8)	18.0, CH_3_
13ʺ	0.84, d (7.0)	20.4, CH_3_	0.90, d (6.8)	20.9, CH_3_
14ʺ	2.13, s	29.9, CH_3_	2.13, s	19.3, CH_3_
15ʺa	3.11, d (12.8)	44.9, CH_2_	2.59, d (12.4)	46.7, CH_2_
15ʺb	1.51, d (12.8)		1.64, d (12.4)	
OH-4	12.65, br s		12.75, br s	
OH-8	2.48, br s			

^a^: Data measured at 400 (^1^H) and 100 (^13^C) MHz in CDCl_3_.

### Simisyzygin B (2)

Yellowish gum; ${\left[\alpha \right]}_{D}^{21}$ −69.2 (*c* 0.39, MeOH); UV (MeOH) *λ*_max_ (log ε) 286 (4.01) nm; CD (MeOH) *λ*_ext_ (Δε) 233.5 (−4.11), 291 (+24.41), 332.5 (−22.37) nm; IR (ATR) *ν*_max_ 3396 (OH), 1727, 1709, 1657 (C = O), 1602 (aromatic ring) cm^−1^; ^1^H NMR and ^13^C NMR data, Table 1; ESIMS *m/z* 521 [M + H]^+;^ HRESIMS *m/z* 543.27176 [M + Na]^+^ (calcd for C_32_H_40_NaO_6_, 543.27226).

### Simisyzygin H1 (8)

Yellowish gum; ${\left[\alpha \right]}_{D}^{21}$ −121.6 (*c* 0.11, MeOH); UV (MeOH) *λ*_max_ (log *ε*) 277 (4.04) nm; IR (ATR) *ν*_max_ 3401 (OH), 1710, 1655 (C = O), 1608 (aromatic ring) cm^−1^; ^1^H NMR and ^13^C NMR data, Table 2; ESIMS *m/z* 519 [M − H]^−^; HRESIMS *m/z* 519.27509 [M − H]^−^ (calcd for C_32_H_39_O_6_, 519.27521).

**Table 2.** ^1^H and ^13^C NMR Data for **8**–**11** in CDCl_3_.

	8^a^		9^b^		10^b^		11^b^	
Position	*δ*_H_ (*J* in Hz)	*δ*_C_, type	*δ*_H_ (*J* in Hz)	*δ*_C_, type	*δ*_H_ (*J* in Hz)	*δ*_C_, type	*δ*_H_ (*J* in Hz)	*δ*_C_, type
2	5.07, t (6.8)	74.2, CH	5.03, dd (10.2, 3.0)	74.7, CH	5.28, dd (10.8, 3.6)	75.1, CH	5.26, dd (10.8, 3.6)	75.2, CH
3a	2.51, d (6.8)	37.4, CH_2_	2.52, dd (17.4, 10.2)	37.3, CH_2_	2.65, dd (17.4, 10.8)	36.1, CH_2_	2.62, dd (17.4, 10.8)	36.2, CH_2_
3b	2.51, d (6.8)		2.46, dd (17.4, 3.0)		2.59, dd (17.4, 3.6)		2.55, dd (17.4, 3.6)	
4		172.4, C		171.5, C		169.7, C		168.7, C
5		194.1, C		195.0, C		194.6, C		195.7, C
6		69.7, C		69.4, C		64.0, C		64.4, C
7		207.6, C		207.8, C		208.4, C		208.5, C
8		77.3, C		78.7, C		80.1, C		80.3, C
9		78.9, C		77.6, C		81.6, C		81.9, C
10		106.3, C		105.9, C		109.6, C		109.6, C
11	1.20, s	6.7, CH_3_	1.21, s	6.8, CH_3_	1.09, s	9.4, CH_3_	1.04, s	9.5, CH_3_
12	1.48, s	23.1, CH_3_	1.38, s	22.9, CH_3_	1.46, s	26.7, CH_3_	1.41, s	26.8, CH_3_
1′		140.2, C		140.5, C		140.4, C		140.7, C
2′, 6′	7.28, m	125.6, CH	7.27, m	125.8, CH	7.42, m	125.7, CH	7.38, m	125.8, CH
3′, 5′	7.32, m	128.4, CH	7.32, m	128.4, CH	7.42, m	128.8, CH	7.38, m	128.8, CH
4′	7.32, m	128.1, CH	7.32, m	128.2, CH	7.37, m	128.5, CH	7.38, m	128.3, CH
1ʺ	3.12, d (9.6)	61.9, CH	4.58, dd (11.3, 3.3)	84.6, CH	5.28, dd (9.0, 6.0)	59.6, CH	5.00, br t (7.2)	124.9, CH
2ʺa	1.96, m	23.9, CH_2_	2.00, m	24.2, CH_2_	2.15, m	26.9, CH_2_	2.25, m	24.6, CH_2_
2ʺb	1.40, m		1.87, m		1.42, m		2.08, m	
3ʺa	1.87, m	35.3, CH_2_	1.54, m	32.2, CH_2_	2.33, ddd (14.4, 5.4, 3.0)	34.9, CH_2_	2.13, m	36.2, CH_2_
3ʺb	1.61, m		1.42, m		1.91, td (14.4, 5.4)		1.91, td (13.2, 5.4)	
4ʺ		45.1, C		45.5, C		48.5, C		48.1, C
5ʺ	3.91, d (11.0)	45.3, CH	3.54, d (10.8)	46.2, CH	2.52, d (11.7)	54.1, CH	2.45, d (12.0)	54.5, CH
6ʺ	4.99, d (11.0)	121.5, CH	5.15, d (10.8)	121.9, CH	4.83, d (11.7)	118.4, CH	4.69, d (12.0)	119.6, CH
7ʺ		146.9, C		149.7, C		150.6, C		147.9, C
8ʺa	2.81, m	26.1, CH_2_	2.67, td (13.5, 1.6)	31.8, CH_2_	2.24, m	25.6, CH_2_	2.11, m	28.4, CH_2_
8ʺb	2.17, m		2.31, ddd (13.5, 6.8, 2.4)		1.97, br dd (14.1, 6.9)		1.97, ddd (13.1, 4.2, 3.2)	
9ʺa	2.17, m	35.8, CH_2_	2.43, m	33.4, CH_2_	2.05, m	37.4, CH_2_	2.20, m	35.9, CH_2_
9ʺb	1.57, m		2.20, td (13.5, 1.6)		1.06, m		1.76, td (13.1, 3.2)	
10ʺ		62.1, C		144.3, C		59.7, C		133.3, C
11ʺ	2.35, sept (6.6)	31.7, CH	2.42, sept (7.2)	35.9, CH	2.22, sept (6.9)	34.7, CH	2.25, sept (7.2)	34.7, CH
12ʺ	1.07, d (6.6)	20.8, CH_3_	1.10, d (7.2)	22.5, CH_3_	1.05, d (6.9)	22.0, CH_3_	1.04, d (7.2)	21.9, CH_3_
13ʺ	1.00, d (6.6)	22.7, CH_3_	1.12, d (7.2)	22.7, CH_3_	1.03, d (6.9)	23.3, CH_3_	1.05, d (7.2)	23.5, CH_3_
14ʺa	1.21, s	20.6, CH_3_	5.24, br s	115.4, CH_2_	1.33, s	16.8, CH_3_	1.56, s	18.0, CH_3_
14ʺb			5.10, br s					
15ʺ	0.85, s	19.0, CH_3_	0.88, s	17.6, CH_3_	1.13, s	19.3, CH_3_	1.04, s	19.8, CH_3_
OH-4	12.91, br s		12.79, br s		12.80, br s		12.71, br s	

^a^: Data measured at 400 (^1^H) and 100 (^13^C) MHz in CDCl_3_;

^b^: Data measured at 600 (^1^H) and 150 (^13^C) MHz in CDCl_3_.

### Simisyzygin H2 (9)

Yellowish gum; ${\left[\alpha \right]}_{D}^{21}$ −83.6 (*c* 0.07, MeOH); UV (MeOH) *λ*_max_ (log *ε*) 290 (3.66) nm; IR (ATR) *ν*_max_ 3401 (OH), 1704, 1655 (C = O), 1604 (aromatic ring) cm^−1^; ^1^H NMR and ^13^C NMR data, Table 2; ESIMS *m/z* 543 [M + Na]^+^; HRESIMS *m/z* 543.27197 [M + Na]^+^ (calcd for C_32_H_40_NaO_6_, 543.27171).

### Simisyzygin H3 (10)

Yellowish gum; ${\left[\alpha \right]}_{D}^{21}$ +24.9 (*c* 0.03, MeOH); UV (MeOH) *λ*_max_ (log *ε*) 290 (3.82) nm; IR (ATR) *ν*_max_ 3479 (OH), 1727, 1657 (C = O), 1602 (aromatic ring) cm^−1^; ^1^H NMR and ^13^C NMR data, Table 2; ESIMS *m/z* 543 [M + Na]^+^; HRESIMS *m/z* 543.27179 [M + Na]^+^ (calcd for C_32_H_40_NaO_6_, 543.27171).

### Simisyzygin H4 (11)

Yellowish gum; ${\left[\alpha \right]}_{D}^{21}$ +12.7 (*c* 0.13, MeOH); UV (MeOH) *λ*_max_ (log *ε*) 285 (3.63) nm; IR (ATR) *ν*_max_ 3458 (OH), 1712, 1659 (C = O), 1603 (aromatic ring) cm^−1^; ^1^H NMR and ^13^C NMR data, Table 2; ESIMS *m/z* 527 [M + Na]^+^; HRESIMS *m/z* 527.27671 [M + Na]^+^ (calcd for C_32_H_42_NaO_5_, 527.27680).

### Simisyzygin I1 (12)

Yellowish gum; ${\left[\alpha \right]}_{D}^{20}$ −8.3 (*c* 1.04, MeOH); UV (MeOH) *λ*_max_ (log *ε*) 285 (3.76) nm; IR (ATR) *ν*_max_ 3428 (OH), 1713, 1657 (C = O), 1604 (aromatic ring) cm^−1^; ^1^H NMR and ^13^C NMR data, Table 3; ESIMS *m/z* 527 [M + Na]^+^; HRESIMS *m/z* 527.27667 [M + Na]^+^ (calcd for C_32_H_40_NaO_5_, 527.27680).

**Table 3.** ^1^H and ^13^C NMR Data for **12**–**14** in CDCl_3_.

	12^a^		13^b^		14^b^	
Position	*δ*_H_ (*J* in Hz)	*δ*_C_, type	*δ*_H_ (*J* in Hz)	*δ*_C_, type	*δ*_H_ (*J* in Hz)	*δ*_C_, type
2	5.21, dd (8.8, 3.7)	73.7, CH	5.22, dd (8.4, 4.0)	73.7, CH	5.21, dd (8.3, 4.1)	73.8, CH
3a	2.76, dd (17.9, 8.8)	35.2, CH_2_	2.79, dd (17.8, 8.4)	35.0, CH_2_	2.74, dd (17.7, 8.3)	34.7, CH_2_
3b	2.66, dd (17.9, 3.7)		2.69, dd (17.8, 4.0)		2.67, dd (17.7, 4.1)	
4		169.1, C		169.1, C		168.5, C
5		195.7, C		195.8, C		196.2, C
6		66.9, C		66.7, C		66.6, C
7		208.0, C		208.1, C		208.7, C
8		77.4, C		77.6, C		78.3, C
9		76.5, C		76.4, C		76.4, C
10		109.3, C		109.0, C		108.3, C
11	1.28, s	7.5, CH_3_	1.26, s	7.2, CH_3_	1.26, s	7.0, CH_3_
12	1.43, s	22.5, CH_3_	1.41, s	22.3, CH_3_	1.40, s	22.3, CH_3_
1′		140.1, C		140.2, C		140.1, C
2′, 6′	7.39, m	126.4, CH	7.41, m	126.5, CH	7.40, m	126.7, CH
3′, 5′	7.39, m	128.7, CH	7.40, m	128.6, CH	7.39, m	128.6, CH
4′	7.36, m	128.4, CH	7.35, m	128.4, CH	7.35, m	128.5, CH
1ʺ		32.2, C		31.4, C		31.6, C
2ʺa	1.88, m	29.1, CH_2_	1.65, dd (8.4, 4.0)	30.7, CH_2_	1.59, m	30.5, CH_2_
2ʺb	1.43, m		1.52, m		1.52, m	
3ʺa	1.23, m	30.4, CH_2_	1.20, m	30.6, CH_2_	1.21, m	30.7, CH_2_
3ʺb	1.23, m		1.20, m		0.87, m	
4ʺ		49.3, C		49.6, C		50.5, C
5ʺ	0.38, d (3.2)	37.4, CH	0.40, d (3.5)	39.4, CH	0.61, d (4.2)	29.7, CH
6ʺ	0.63, t (3.2)	24.8, CH	0.58, dd (3.5, 1.8)	23.0, CH	1.04, m	25.1, CH
7ʺ	1.12, m	42.6, CH	1.26, m	41.1, CH	1.36, m	42.1, CH
8ʺa	1.34, m	25.2, CH_2_	1.11, m	19.0, CH_2_	1.21, m	19.0, CH_2_
8ʺb	0.80, m		1.05, m		0.65, m	
9ʺa	1.54, m	31.1, CH_2_	1.30, m	28.8, CH_2_	1.29, m	30.6, CH_2_
9ʺb	0.48, m		1.20, dd (9.6, 4.8)		1.16, m	
10ʺ	1.67, m	30.3, CH	1.84, ddd (6.8, 4.8, 2.4)	28.9, CH	1.95, q (6.8)	27.6, CH
11ʺ	1.64, br sept (6.8)	33.2, CH	1.60, m	33.5, CH	1.61, m	32.1, CH
12ʺ	0.88, d (6.8)	19.2, CH_3_	0.90, d (6.8)	19.3, CH_3_	0.86, d (6.9)	19.6, CH_3_
13ʺ	0.92, d (6.8)	20.3, CH_3_	0.92, d (6.8)	19.8, CH_3_	1.00, d (6.9)	22.5, CH_3_
14ʺ	0.86, d (6.4)	19.3, CH_3_	0.96, d (6.8)	17.7, CH_3_	0.80, d (6.8)	19.1, CH_3_
15ʺa	2.50, d (12.8)	46.7, CH_2_	2.48, d (12.6)	45.9, CH_2_	2.55, d (12.9)	44.5, CH_2_
15ʺb	1.51, d (12.8)		1.40, d (12.6)		1.34, d (12.9)	
OH-4	12.80, br s		12.80, br s		12.54, br s	

^a^: Data measured at 400 (^1^H) and 100 (^13^C) MHz in CDCl_3_;

^b^: Data measured at 600 (^1^H) and 150 (^13^C) MHz in CDCl_3_.

### Simisyzygin I2 (13)

Colorless gum; ${\left[\alpha \right]}_{D}^{24}$ +10.7 (*c* 0.13, MeOH); UV (MeOH) *λ*_max_ (log *ε*) 285 (3.96) nm; IR (ATR) *ν*_max_ 3464 (OH), 1713, 1661 (C = O), 1610 (aromatic ring) cm^−1^; ^1^H NMR and ^13^C NMR data, Table 3; ESIMS *m/z* 527 [M + Na]^+^; HRESIMS *m/z* 527.27650 [M + Na]^+^ (calcd for C_32_H_40_NaO_5_, 527.27680).

### Simisyzygin I3 (14)

Colorless gum; ${\left[\alpha \right]}_{D}^{20}$ −358.0 (*c* 0.03, MeOH); UV (MeOH) *λ*_max_ (log *ε*) 292 (4.42) nm; IR (ATR) *ν*_max_ 3446 (OH), 1711, 1660 (C = O), 1605 (aromatic ring) cm^−1^; ^1^H NMR and ^13^C NMR data, Table 3; ESIMS *m/z* 527 [M + Na]^+^; HRESIMS *m/z* 527.27661 [M + Na]^+^ (calcd for C_32_H_40_NaO_5_, 527.27680).

### Simisyzygin J1 (15)

Yellowish gum; ${\left[\alpha \right]}_{D}^{25}$ −73.5 (*c* 0.08, MeOH); UV (MeOH) *λ*_max_ (log *ε*) 290 (3.96) nm; IR (ATR) *ν*_max_ 3453 (OH), 1713, 1656 (C = O), 1603 (aromatic ring) cm^−1^; ^1^H NMR and ^13^C NMR data, Table 4; ESIMS *m/z* 527 [M + Na]^+^; HRESIMS *m/z* 527.27705 [M + Na]^+^ (calcd for C_32_H_40_NaO_5_, 527.27680).

**Table 4.** ^1^H and ^13^C NMR Data for **15**–**18** in CDCl_3_.

	15^a^		16^b^		17^b^		18^b^	
Position	*δ*_H_ (*J* in Hz)	*δ*_C_, type	*δ*_H_ (*J* in Hz)	*δ*_C_, type	*δ*_H_ (*J* in Hz)	*δ*_C_, type	*δ*_H_ (*J* in Hz)	*δ*_C_, type
2	5.17, dd (9.8, 3.3)	74.1, CH	5.19, dd (9.6, 3.6)	73.9, CH	5.22, dd (8.0, 4.1)	73.7, CH	5.17, dd (9.2, 3.6)	74.0, CH
3a	2.74, dd (17.9, 9.8)	35.9, CH_2_	2.77, dd (18.0, 9.6)	35.4, CH_2_	2.76, dd (18.0, 8.0)	34.5, CH_2_	2.72, dd (17.9, 9.2)	35.7, CH_2_
3b	2.64, dd (17.9, 3.3)		2.66, dd (18.0, 3.6)		2.68, dd (18.0, 4.1)		2.63, dd (17.9, 3.6)	
4		169.6, C		169.7, C		168.6, C		169.5, C
5		195.8, C		195.8, C		196.0, C		195.8, C
6		68.0, C		68.0, C		65.0, C		68.4, C
7		208.1, C		208.2, C		209.6, C		208.9, C
8		77.7, C		77.7, C		78.1, C		77.2, C
9		76.6, C		76.5, C		76.2, C		76.5, C
10		109.3, C		109.4, C		108.3, C		109.0, C
11	1.14, s	7.0, CH_3_	1.14, s	7.0, CH_3_	1.34, s	8.4, CH_3_	1.05, s	8.3, CH_3_
12	1.45, s	22.7, CH_3_	1.44, s	22.6, CH_3_	1.36, s	22.2, CH_3_	1.40, s	22.4, CH_3_
1′		140.2, C		140.1, C		140.1, C		140.3, C
2′, 6′	7.38, m	126.3, CH	7.39, m	126.5, CH	7.39, m	126.7, CH	7.40, m	126.4, CH
3′, 5′	7.37, m	128.7, CH	7.39, m	128.7, CH	7.39, m	128.6, CH	7.39, m	128.7, CH
4′	7.33, m	128.4, CH	7.33, m	128.5, CH	7.33, m	128.4, CH	7.35, m	128.4, CH
1ʺ	1.52, m	39.0, CH	2.15, m	36.4, CH		41.1, C		41.7, C
2ʺa	1.86, dq (13.2, 3.0)	28.5, CH_2_	1.55, m	22.3, CH_2_	1.43, dd (11.7, 5.7)	40.5, CH_2_	1.47, m	40.6, CH_2_
2ʺb	1.35, m		1.55, m		1.42, dd (5.7, 4.2)		1.46, m	
3ʺa	1.38, m	30.2, CH_2_	1.31, m	30.2, CH_2_	1.74, m	33.4, CH_2_	1.89, m	42.2, CH_2_
3ʺb	1.38, m		1.31, m		1.20, dd (11.7, 5.4)		1.60, m	
4ʺ		42.7, C		42.6, C		50.7, C		51.8, C
5ʺ	4.92, br s	125.8, CH	5.02, br s	127.9, CH	1.52, m	52.6, CH	1.73, m	48.5, CH
6ʺ		144.2, C		141.8, C	1.92, d (4.8)	48.3, CH	1.77, d (5.4)	50.0, CH
7ʺ	1.56, m	51.8, CH	1.52, m	51.0, CH	1.27, m	55.0, CH	1.34, m	55.0, CH
8ʺa	1.99, m	25.5, CH_2_	1.60, m	22.4, CH_2_	1.82, m	28.3, CH_2_	1.86, m	28.4, CH_2_
8ʺb	1.20, m		1.60, m		1.74, m		1.67, m	
9ʺa	1.42, m	29.8, CH_2_	1.76, m	28.9, CH_2_	1.72, dd (16.2, 7.2)	24.6, CH_2_	1.65, m	27.7, CH_2_
9ʺb	1.28, m		1.32, m		1.48, m		1.60, m	
10ʺ	0.98, m	41.4, CH	1.88, m	32.9, CH	1.98, dd (9.0, 7.2)	43.6, CH	2.14, m	47.1, CH
11ʺ	1.74, m	26.4, CH	1.78, m	27.0, CH	1.29, m	29.1, CH	1.29, m	29.7, CH
12ʺ	0.88, d (6.6)	20.9, CH_3_	0.89, d (6.6)	21.4, CH_3_	0.86, d (6.3)	20.8, CH_3_	0.80, d (6.3)	20.8, CH_3_
13ʺ	0.75, d (6.6)	22.1, CH_3_	0.74, d (6.6)	22.4, CH_3_	0.93, d (6.3)	21.9, CH_3_	0.86, d (6.3)	21.9, CH_3_
14ʺ	0.92, d (6.0)	19.9, CH_3_	0.76, d (6.6)	14.3, CH_3_	0.89, s	21.9, CH_3_	0.91, s	22.1, CH_3_
15ʺa	2.74, d (12.6)	45.4, CH_2_	2.72, d (12.6)	44.9, CH_2_	2.44, d (12.6)	44.3, CH_2_	2.88, d (12.9)	41.2, CH_2_
15ʺb	1.63, d (12.6)		1.62, d (12.6)		1.00, d (12.6)		1.46, d (12.9)	
OH-4	12.80 br s		12.80, br s		12.65, br s		12.58, br s	

^a^: Data measured at 400 (^1^H) and 100 (^13^C) MHz in CDCl_3_;

^b^: Data measured at 600 (^1^H) and 150 (^13^C) MHz in CDCl_3_.

### Simisyzygin J2 (16)

Yellowish gum; ${\left[\alpha \right]}_{D}^{24}$ −26.8 (*c* 0.02, MeOH); UV (MeOH) *λ*_max_ (log *ε*) 297.5 (3.37) nm; IR (ATR) *ν*_max_ 3625 (OH), 1722, 1657 (C = O), 1608 (aromatic ring) cm^−1^; ^1^H NMR and ^13^C NMR data, Table 4; ESIMS *m/z* 527 [M + Na]^+^; HRESIMS *m/z* 527.27686 [M + Na]^+^ (calcd for C_32_H_40_NaO_5_, 527.27680).

### Simisyzygin K1 (17)

Colorless gum; ${\left[\alpha \right]}_{D}^{24}$ −17.6 (*c* 0.04, MeOH); UV (MeOH) *λ*_max_ (log *ε*) 297.5 (3.49) nm; IR (ATR) *ν*_max_ 3424 (OH), 1739, 1689 (C = O), 1622 (aromatic ring) cm^−1^; ^1^H NMR and ^13^C NMR data, Table 4; ESIMS *m/z* 527 [M + Na]^+^; HRESIMS *m/z* 527.27686 [M + Na]^+^ (calcd for C_32_H_40_NaO_5_, 527.27680).

### Simisyzygin K2 (18)

Colorless gum; ${\left[\alpha \right]}_{D}^{20}$ −155.2 (*c* 0.03, MeOH); UV (MeOH) *λ*_max_ (log *ε*) 292 (4.00) nm; IR (ATR) *ν*_max_ 3449 (OH), 1723, 1659 (C = O), 1606 (aromatic ring) cm^−1^; ^1^H NMR and ^13^C NMR data, Table 4; ESIMS *m/z* 527 [M + Na]^+^; HRESIMS *m/z* 527.27661 [M + Na]^+^ (calcd for C_32_H_40_NaO_5_, 527.27680).

### Preparation of human neutrophils

Blood was collected from healthy human donors (20–35 years old) by drawing approximately 50 mL from the cubital vein using sterile vacuum tubes. Neutrophils were isolated with a standard method of dextran sedimentation prior to centrifugation in a Ficoll Hypaque gradient and hypotonic lysis of erythrocytes (Böyum et al. 1991). Purified neutrophils that contained > 98% viable cells, as determined by the trypan blue exclusion method, were resuspended in a calcium (Ca^2+^)-free Hanks’ Balanced Salt Solution (HBSS; Gibco, 14175095) buffer at pH 7.4, and were maintained at 4 °C before use.

### Measurement of superoxide anion generation and elastase release

For superoxide anion detection, neutrophil suspensions (6 × 10^5^ cells/mL) containing 0.6 mg/mL ferricytochrome c (cytochrome c from horse heart; Sigma-Aldrich, C2506) in HBSS supplemented with 1 mM CaCl_2_·2H_2_O (calcium chloride dihydrate; Sigma-Aldrich, 21097) and 1 mM MgCl_2_·6H_2_O (magnesium chloride hexahydrate; Sigma-Aldrich, M2670) were treated with the various compounds or 0.1% DMSO (Sigma-Aldrich, D1435) as the vehicle control for 2 min at 37 °C, followed by treatment with 1 μg/mL cytochalasin B (CB; Sigma-Aldrich, C6762) for 3 min. Cells were then activated with 0.1 μM *N*-formyl-methionine-leucyl-phenylalanine (fMLP; Sigma-Aldrich, 47729) as a stimulant for approximately 10 min. Absorbance was measured at 550 nm using a UV spectrophotometer. For elastase release experiments, neutrophil suspensions (6 × 10^5^ cells/mL) containing 100 μM elastase substrate, MeO-Suc-Ala-Ala-Pro-Val-*p*-nitroanilide (Millipore, 454454), in HBSS supplemented with 1 mM CaCl_2_·2H_2_O and 1 mM MgCl_2_·6H_2_O, were preheated for 5 min to reach 37 °C. The various compounds or 0.1% DMSO were then added for 2 min, followed by treatment with 0.5 μg/mL CB for 3 min. Neutrophils were then stimulated with 0.1 μM fMLP for approximately 10 min. Finally, absorbance was measured at 405 nm using a UV spectrophotometer. The percentage inhibition (Inh%) was calculated using the formula: Inh% = [(Ind−DMSO)−(Cpd−DMSO)]/(Ind−DMSO) × 100%. Ind (induced group) is the absorbance of the 0.1% DMSO-treated group following fMLP/CB stimulation, DMSO is the absorbance of the 0.1% DMSO-treated group without fMLP/CB stimulation, and Cpd (compound-treated group) is the absorbance of the test compound-treated group following fMLP/CB stimulation. Statistical analysis was performed using GraphPad Prism 9 (version 9.0, GraphPad Software Inc., CA, USA). Data are presented as mean ± standard error of the mean (SEM). Student’s t-test was applied to assess statistical significance, with *p* < 0.05 considered statistically significant. The 50% inhibitory concentration (IC_50_) was calculated from the dose-response curve obtained by plotting the percentage of inhibition versus concentrations (linear function, Microsoft Office, Redmond, WA, U.S) (Gamal El-Din et al. 2022). All experiments were conducted in triplicate. LY294002 (Sigma-Aldrich, 440204) served as the positive control (Hwang et al. 2006).

## Results and discussion

### Structure elucidation

Compound **1** crystallized as colorless prisms. The HRESIMS revealed a sodium adduct ion peak at *m/z* 543.27286 [M + Na]^+^, manifesting its molecular formula to be C_32_H_40_O_6_ with thirteen degrees of unsaturation. IR absorption at 3401 cm^−1^ (hydroxyl groups), at 1724 and 1711 cm^−1^ (carbonyl groups), at 1659 cm^−1^ (*α*,*β*-unsaturated carbonyl group), and at 1610 cm^−1^ (aromatic ring). The ^1^H NMR data (Table 1) of **1** showed three singlet methyl groups (*δ*_H_ 1.16, 1.46, and 2.13), an isopropyl group with methyl groups [*δ*_H_ 0.84 and 0.87 (each 3H, d, *J* = 7.0 Hz)] coupling to a methine [*δ*_H_ 1.76 (1H, septd, *J* = 7.0, 2.8 Hz)], a hydroxyl proton [*δ*_H_ 2.48 (1H, br s, D_2_O exchangeable)], a deshielded oxymethine [*δ*_H_ 5.77 (1H, dd, *J* = 9.2, 3.6 Hz)], a monosubstituted benzene [*δ*_H_ 7.36 (1H, m) and 7.39 (4H, m)], and an intermolecular hydrogen bond [*δ*_H_ 12.65 (1H, br s)]. Comprehensive analysis of ^13^C NMR (Table 1), DEPT, and HSQC spectra showed five methyl groups (*δ*_C_ 7.6, 18.0, 20.4, 22.5, and 29.9); six sp^3^ methylenes (*δ*_C_ 24.1, 29.2, 35.7, 38.1, 42.5, and 44.9); eleven methines, including six sp^3^ (*δ*_C_ 23.7, 28.0, 30.9, 32.6, 47.0 and 74.0) and five sp^2^ [*δ*_C_ 126.4 (overlapped), 128.5, and 128.7 (overlapped)]; and ten quaternary carbons, including four sp^3^ carbons (*δ*_C_ 49.7, 68.7, 76.4, and 77.2) and six sp^2^ carbons—three olefinic (*δ*_C_ 109.0, 140.1, 169.7) and three carbonyl (*δ*_C_ 195.5, 208.4, and 209.4).

The presence of a monosubstituted aromatic ring was observed by typical ^1^H NMR and ^13^C NMR signals [*δ*_H_ 7.36 (1H, m) and 7.39 (4H, m); *δ*_C_ 126.4 (overlapped), 128.5, 128.7 (overlapped), and 140.1], further supported by HMBC correlations (Figure 2) from H-4′ to C-2′/C-3′, and H-3′ to C-1′. The HMBC correlations from H-11 to C-5/C-6/C-7 positioned a methyl group on a sp^3^ quaternary carbon (C-6, *δ*_C_ 68.7), displaying its resonance chemical shift consistent with its *α*-position of two carbonyl groups (C-5, *δ*_C_ 195.5, and C-7, *δ*_C_ 208.4). The ketone carbon C-5 (*δ*_C_ 195.5) exhibited an upfield shift, presumably influenced by electron delocalization from the conjugated *α*,*β*-unsaturated system connected to olefinic centers (C-4, *δ*_C_ 169.7, and C-10, *δ*_C_ 109.0). Notably, the unusually downfield C-4 resonance (*δ*_C_ 169.7) compared to typical olefinic carbons indicated a hydroxylated olefinic carbon forming an enol configuration. This was further supported by the extremely downfield OH-4 proton (*δ*_H_ 12.65), which participated in an intramolecular hydrogen bond with the C-5 carbonyl oxygen. A close comparison of NMR chemical shifts and corresponding HMBC correlation patterns in the previous study confirmed the remarkable similarity of flavanone moieties, that the newly isolated hybrid possessed the same 5,7-dioxo-6,8-dimethylflavanone moiety attached to a sesquiterpenoid unit (Yang et al. 2025).

**Figure 2.**
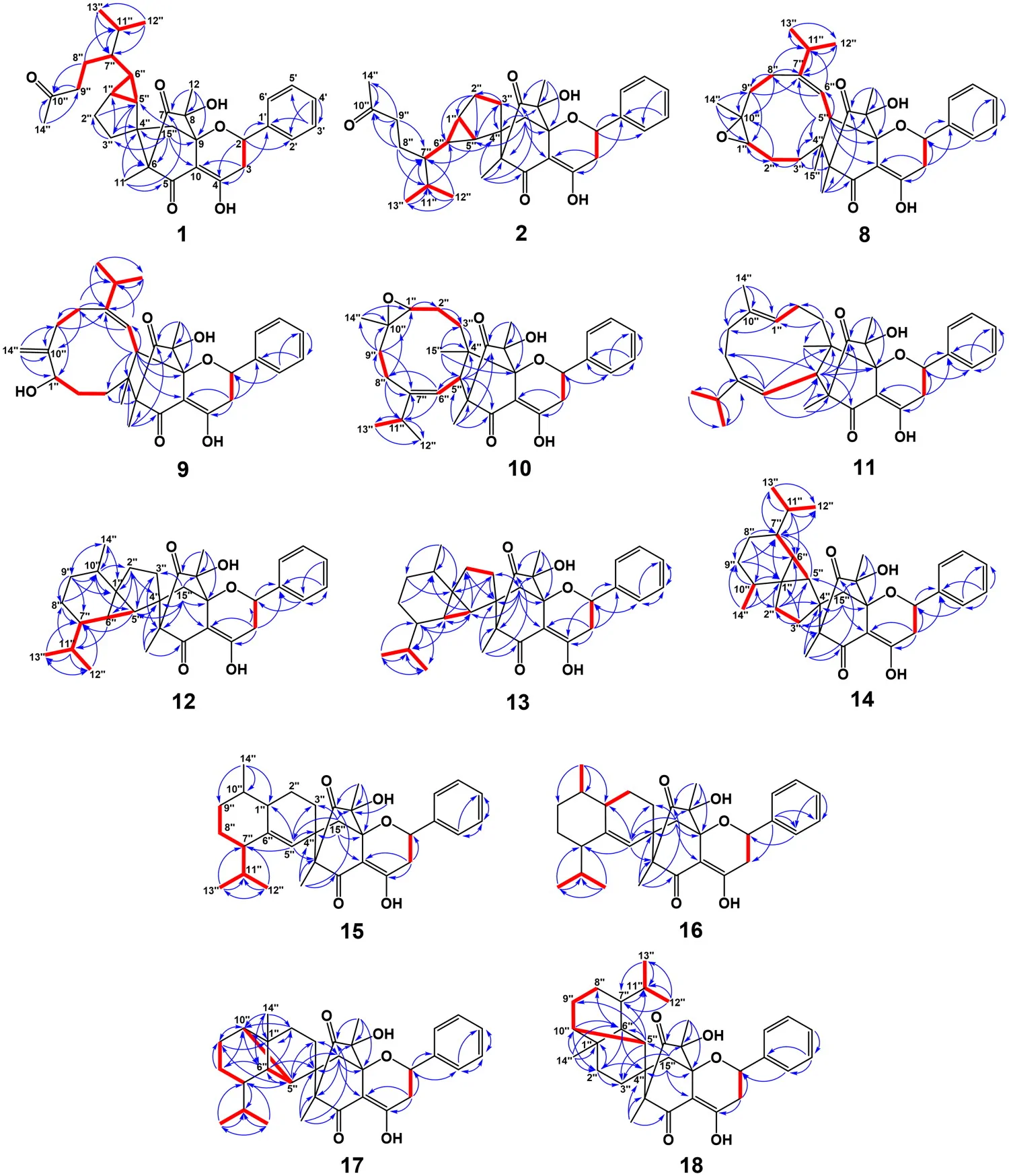
Key HMBC (→) and COSY (─) correlations for **1**–**2**, and **8**–**18**.

Subsequently, COSY correlations of H-1ʺ/H-5ʺ, H-1ʺ/H-6ʺ, H-5ʺ/H-6ʺ, H-6ʺ/H-7ʺ, H-7ʺ/H-8ʺ, and H-8ʺ/H-9ʺ revealed a C-1ʺ—C-5ʺ—C-6ʺ—C-7ʺ—C-8ʺ—C-9ʺ fragment containing a C-1ʺ—C-5ʺ—C-6ʺ cyclopropane ring, confirmed by the characteristic upfield shifts of H-5ʺ (*δ*_H_ 0.91), H-6ʺ (*δ*_H_ 0.34), and H-7ʺ (*δ*_H_ 0.45). HMBC correlations from H-2ʺ to C-5ʺ, H-3ʺ to C-1ʺ/C-2ʺ/C-4ʺ, and H-5ʺ to C-3ʺ established a bicyclo[3.1.0]hexane system with C-1ʺ and C-5ʺ as bridgehead carbons. COSY correlations of H-11ʺ/H-12ʺ and H-11ʺ/H-13ʺ, along with HMBC correlations from H-12ʺ to C-7ʺ/C-11ʺ/C-13ʺ, H-13ʺ to C-7ʺ/C-11ʺ, and H-11ʺ to C-7ʺ, confirmed an isopropyl group attached at C-7ʺ. HMBC correlations from the singlet H_3_-14ʺ to C-9ʺ/C-10ʺ placed CH_3_-14ʺ adjacent to carbonyl C-10ʺ (*δ*_C_ 209.4), forming a 5,6-dimethylheptan-2-one side chain linked to the bicyclo[3.1.0]hexane moiety at C-6ʺ. These features indicated a *seco*-cubebene-type scaffold with an opened cyclohexanone structure.

Finally, key HMBC correlations from H_3_-11 to C-4ʺ, H-5ʺ to C-6 and H-15ʺ to C-3ʺ/C-4ʺ/C-5ʺ established a spiro junction at C-4ʺ, connecting the sesquiterpenoid and flavanone moieties to form a spiro[5.5]undecane configuration in the structure. The flavonoid stereochemistry was assigned as 2*S*,6*S*,8*R*,9*S* based on the X-ray structure of **3** (Yang et al. 2025), which shared an identical flavonoid moiety and likely followed a similar biosynthetic pathway. NOESY correlations (Figure 3) between H-5ʺ/H-7ʺ and H-1ʺ/H-7ʺ indicated that H-1ʺ, H-5ʺ, and H-7ʺ were cofacial, establishing a *cis*-fused bicyclo[3.1.0]hexane, while H-6ʺ was on the opposite face. Notably, based on the pattern of spiro-ring formation, two distinct stereostructures, sA-type and sB-type, were proposed for discussion (Figure 4). NOESY correlations between H-15ʺa/H-5ʺ and H-15ʺb/H-3ʺb confirmed an sA-type configuration for **1**.

**Figure 3.**
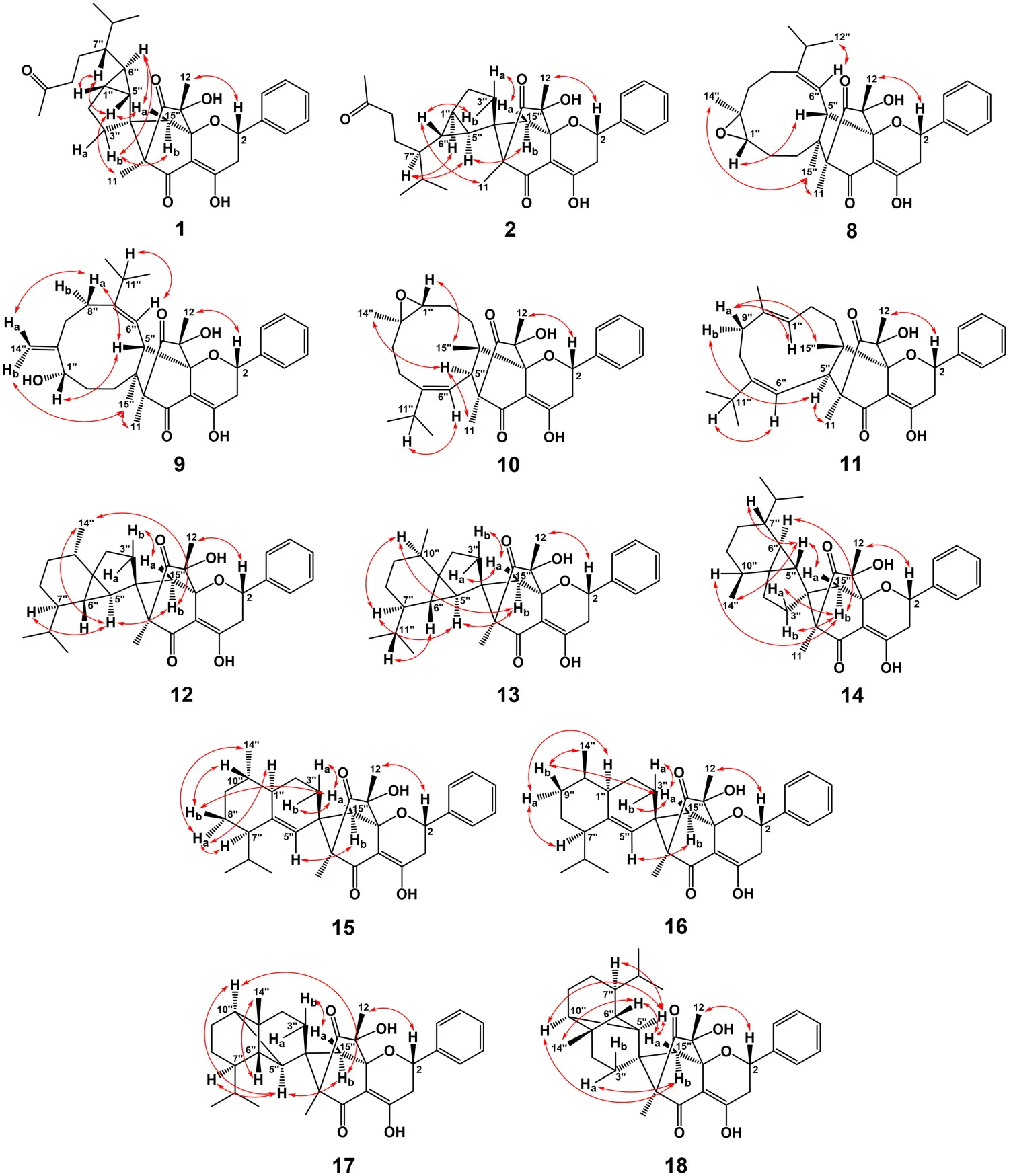
Key NOESY (↔) correlations for **1**–**2**, and **8**–**18**.

**Figure 4.**
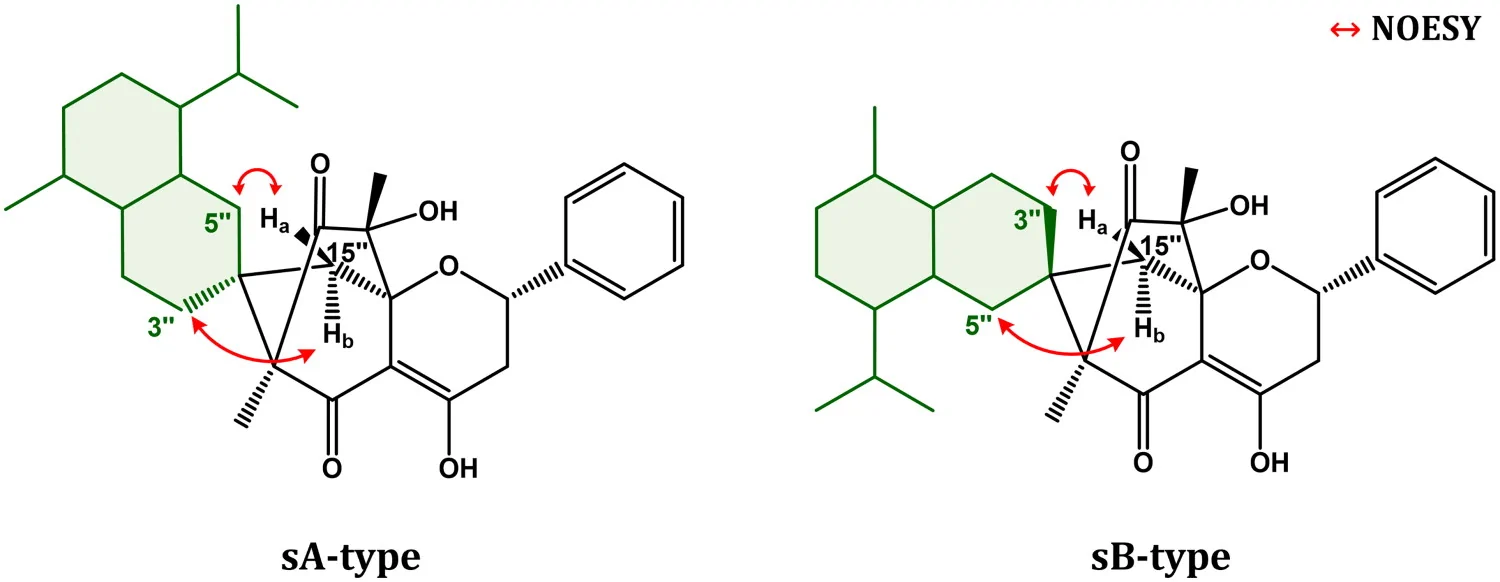
Two spiro-connection patterns, designated as sA and sB types, were identified for compounds **1**–**2**, and **12**–**18** based on characteristic NOESY correlations.

While the planar structure of **1** matched that of syzygioblanes A and B (Koga et al. 2024a), X-ray crystallographic analysis using Cu Kα radiation (Figure 5) revealed a different absolute configuration (1ʺ*R*,2*S*,4ʺ*R*,5ʺ*R*,6*S*,6ʺ*R*,7ʺ*R*,8*R*,9*S*), establishing **1** as a diastereomer of the known compounds. Thus, compound **1** was elucidated and named simisyzygin A.

**Figure 5.**
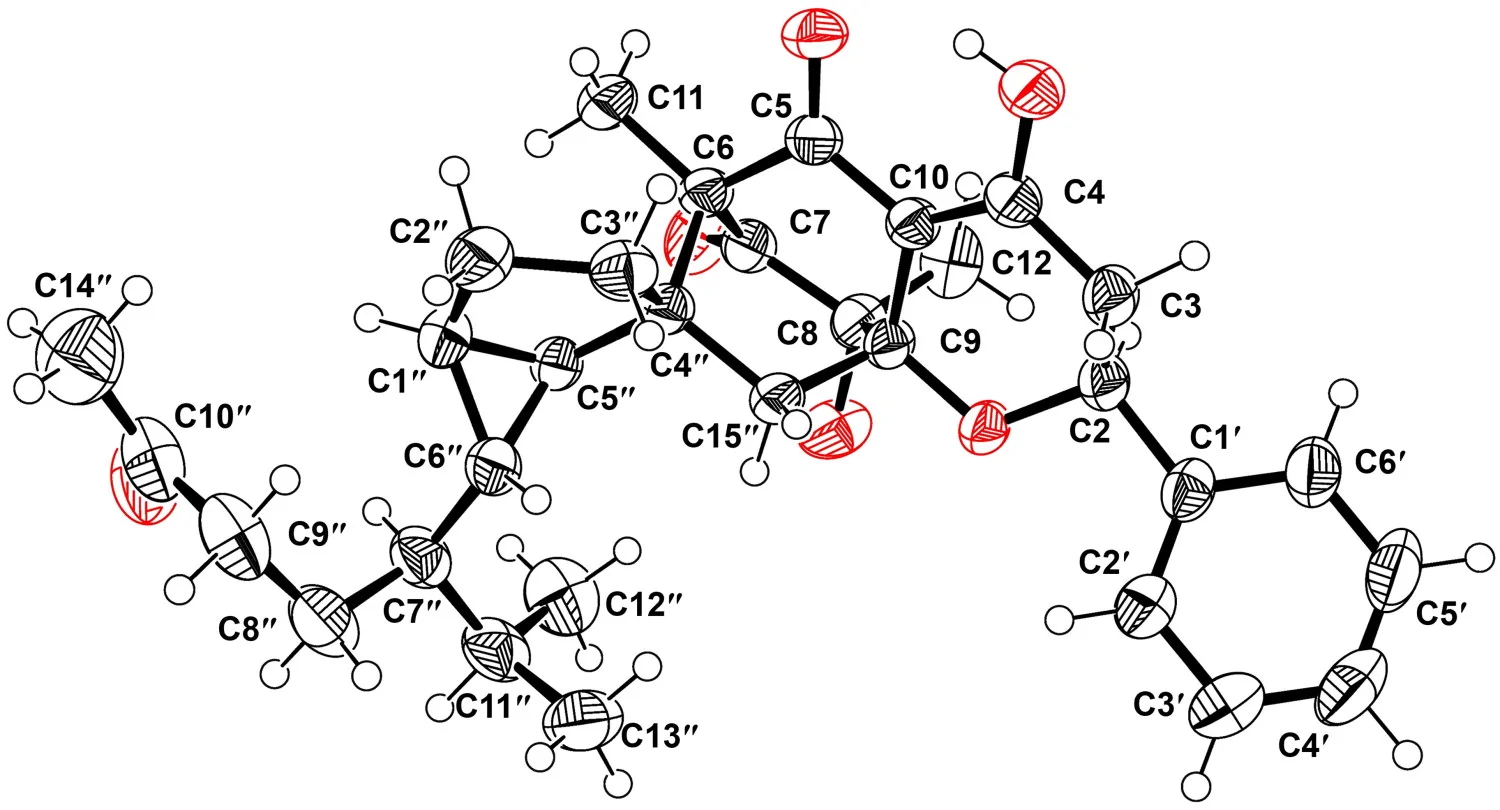
ORTEP view of compound **1**.

Compound **2** exhibited NMR spectra (Table 1) closely resembling those of **1**, with the primary distinction observed in NOESY correlations. While **1** displayed correlations between H-15ʺa/H-5ʺ and H-15ʺb/H-3ʺb, compound **2** showed correlations between H-15ʺa/H-3ʺa and H-15ʺb/H-5ʺ (Figure 3), indicating an sB-type spiro configuration at C-4ʺ (Figure 4). The CD spectrum of **1** closely matched that of **2** (Figure 6), supporting analogous absolute stereochemistry of **2**. Thus, compound **2** was assigned as 1ʺ*R*,2*S*,4ʺ*S*,5ʺ*R*,6*S*,6ʺ*R*,7ʺ*R*,8*R*,9*S* and named simisyzygin B.

**Figure 6.**
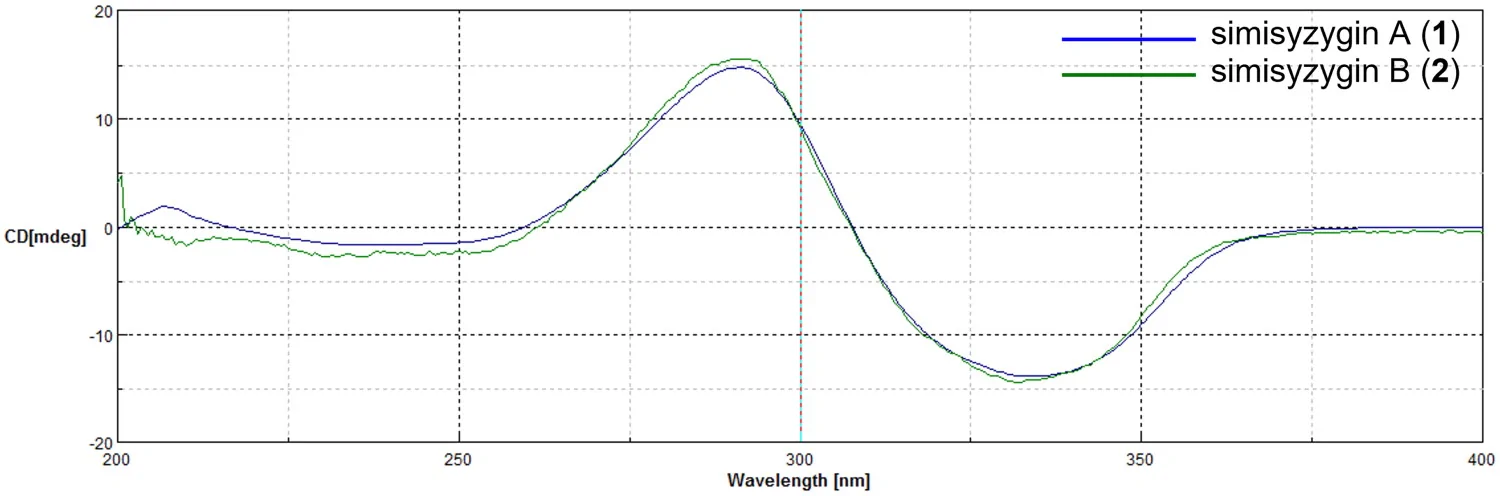
CD spectrum of **1** and **2**.

Compound **8** was obtained as yellowish gum and showed a molecular formula of C_32_H_40_O_6_ by HRESIMS (*m/z* 519.27509 [M − H]^−^). Comparison of ^1^H and ^1^³C NMR spectra (Tables 1 and 2) and HMBC/COSY correlations (Figure 2) between compounds **8** and **1** revealed that **8** possessed the same flavanone moiety but differed in its sesquiterpenoid moiety, as evidenced by the replacement of a methyl group at C-15ʺ (*δ*_C_ 19.0) in **8** with a downfield-shifted methylene at C-15ʺ (*δ*_C_ 44.9) in **1**. The presence of a cyclodecane core was indicated by COSY correlations of H-1ʺ/H-2ʺ/H-3ʺ, H-5ʺ/H-6ʺ, and H-8ʺ/H-9ʺ, along with HMBC correlations from H-5ʺ to C‑3ʺ/C-4ʺ/C-6ʺ/C-7ʺ, H-6ʺ to C-8ʺ, and H-9ʺ to C‑1ʺ/C-7ʺ/C-10ʺ. An endocyclic double bond was suggested by the characteristic chemical shifts of C-6ʺ (*δ*_C_ 121.5) and C-7ʺ (*δ*_C_ 146.9), while an epoxide ring was formed between C-1ʺ (*δ*_C_ 61.9) and C-10ʺ (*δ*_C_ 62.1). The isopropyl substituent was positioned at C-7ʺ, as confirmed by COSY correlations of H-7ʺ/H-11ʺ, H-11ʺ/H-12ʺ and H-11ʺ/H-13ʺ, and HMBC correlations from H-11ʺ and H-12ʺ to C-7ʺ, and from H-6ʺ and H-8ʺ to C-11ʺ. A tertiary methyl group (CH_3_-15ʺ) was assigned at C-4ʺ based on HMBC correlations from H_3_-15ʺ to C-6/C-3ʺ/C-4ʺ, while a secondary methyl group (CH_3_-14ʺ) was placed at C-10ʺ based on HMBC correlations from H_3_-14ʺ to C-1ʺ/C-9ʺ/C-10ʺ. These spectral features collectively established a germacrene-type sesquiterpenoid skeleton.

Additionally, the sesquiterpenoid moiety was ring-fused to the flavanone structure through key HMBC correlations from H-5ʺ to C-8/C-9/C-10, H-11 to C-4ʺ, and H-15ʺ to C-6. The ring fusion connection pattern could be divided into rA-type and rB-type based on their connectivity arrangements (Figure 7). In compound **8**, where C-4ʺ connected to C-6 and C-5ʺ connected to C-9, the compound was therefore classified as rA-type. Based on the molecular formula, a hydroxyl group at C-8 (*δ*_C_ 77.3) accounted for the remaining oxygen atom.

**Figure 7.**
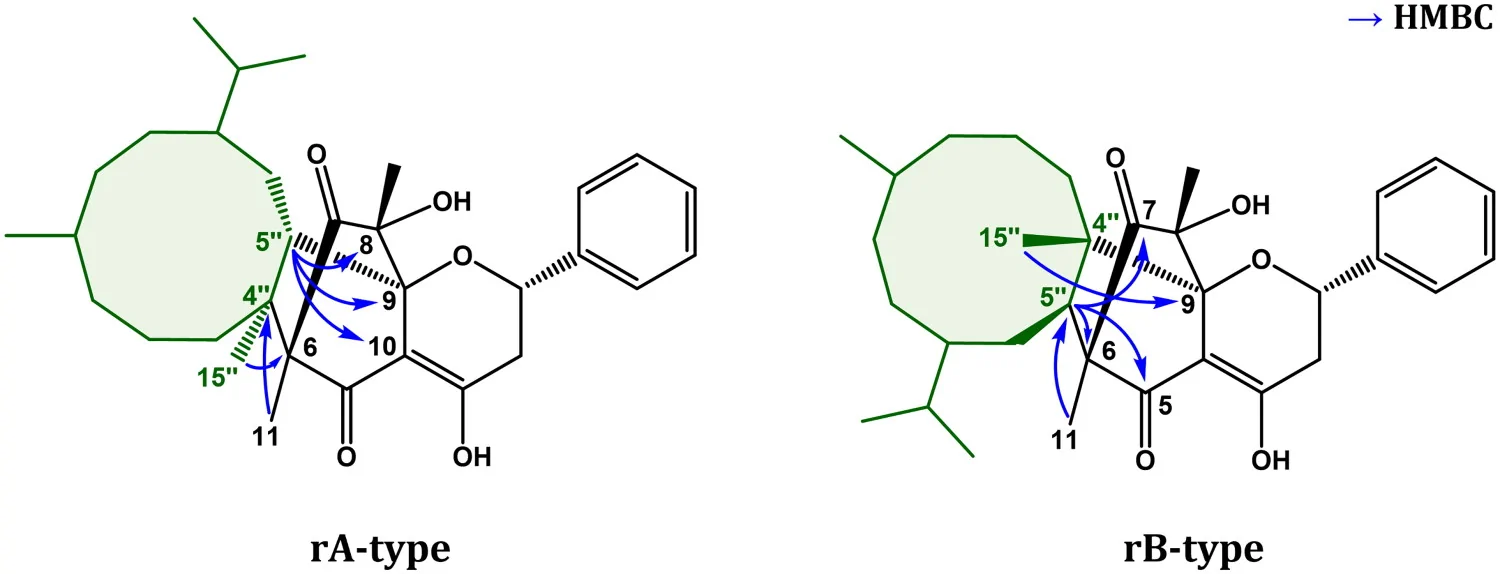
Two ring fusion patterns, designated as rA and rB types, were identified for compounds **8**–**11** based on characteristic HMBC correlations.

The planar structure of **8** featured a rare tricyclic framework comprising a bicyclo[8.4.0]tetradecane with C-4ʺ and C-5ʺ as bridgehead carbons, fused to a bicyclo[2.2.2]octane unit where C-6 and C-9 serve as bridgehead carbons. It was determined to share the same planar structure as **3** but with stereochemical differences in the terpene portion. NOESY correlations between H-6ʺ/H_3_-12ʺ confirmed the *E*-configuration of the C-6ʺ/C-7ʺ double bond (Figure 3). Moreover, H_3_-11/H_3_-15ʺ and H_3_-14ʺ/H_3_-15ʺ correlations indicated CH_3_-11, CH_3_-14ʺ, and CH_3_-15ʺ in *α*-orientation, while H-1ʺ/H-5ʺ correlations indicated these protons in *β*-orientation. Therefore, compound **8** differed from **3** only in the stereochemistry at positions 1ʺ and 10ʺ. Accordingly, the structure of **8** was designated simisyzygin H1.

Compound **9** shared the same molecular formula as **8** but exhibited subtle differences in NMR data (Table 2), indicating distinct configurations between the two compounds. Furthermore, compound **9** was determined to have the same planar structure as **4** based on comprehensive 2D NMR spectroscopic analysis, including HMQC, HMBC and COSY experiments (Figure 2), which confirmed the presence of a germacrene-type sesquiterpenoid portion characterized by a hydroxyl group at C-1ʺ and a terminal double bond at C-10ʺ. However, the key NOESY interaction between H-1ʺ/H-5ʺ indicated an *S* configuration at C-1ʺ in **9** (Figure 3), which contrasted with the *R* configuration in **4** established by the H-1ʺ/H_3_-15ʺ correlation (Yang et al. 2025). This revealed that compound **9** represented a stereoisomer of **4** with the opposite configuration at the single chiral center (C-1ʺ). Therefore, the structure of **9** was constructed and named simisyzygin H2.

Compound **10** was isolated as yellowish gum, and the molecular formula was established as C_32_H_40_O_6_. The ^1^H and ^13^C NMR data of **10** were nearly identical to those of **8** (Table 2). The signature resonances at *δ*_C_ 59.6 (C-1ʺ) and 59.7 (C-10ʺ), together with relevant HMBC correlations (Figure 2), indicated the presence of a 1ʺ,10ʺ-epoxide-germacrene moiety in the sesquiterpenoid portion, analogous to that in compounds **3** and **8**. Notably, key HMBC correlations from H_3_-15ʺ to C-9 and H_3_-11 to C-5ʺ demonstrated a distinct connectivity pattern with C-4ʺ―C-9 and C-5ʺ―C-6 of the sesquiterpenoid unit, which differed from that observed in **3** and **8** with C-4ʺ―C-6 and C-5ʺ―C-9, and was therefore classified as rB-type (Figure 7). The structural differences were further corroborated by the positive optical rotation of compound **10** {${\left[\alpha \right]}_{D}^{21}$ +24.9 (*c* 0.03, MeOH)}, which contrasted with the negative optical rotation observed for **3** {${\left[\alpha \right]}_{D}^{21}$ −190.8 (*c* 0.08, MeOH)} and **8** {${\left[\alpha \right]}_{D}^{21}$ −121.6 (*c* 0.11, MeOH)}, collectively revealing a different structural arrangement between the sesquiterpenoid and flavonoid moieties. The relative stereochemistry of the sesquiterpenoid moiety in **10** was established through NOESY experiments (Figure 3). Key NOESY correlations indicated that H-1ʺ and CH_3_-15ʺ were positioned on the same face of the molecule, while H-5ʺ and CH_3_-14ʺ were located on the opposite face, revealing a stereochemical arrangement identical to that of **3**. Based on comprehensive spectroscopic analysis, the structure of compound **10** was elucidated and designated as simisyzygin H3.

Structural elucidation via 1D and 2D NMR spectroscopic analysis indicated that compound **11** possessed the same sesquiterpenoid moiety as **5**, characterized by the presence of an internal double bond with olefinic carbon signals at *δ*_C_ 124.9 (C-1′′) and *δ*_C_ 133.3 (C-10′′) (Table 2; Figures 2 and 3). Key HMBC correlations from H_3_-15ʺ to C-9 and H_3_-11 to C-5ʺ established that the sesquiterpenoid unit was connected to the flavonoid core via an rB-type linkage (C-4ʺ―C-9 and C-5ʺ―C-6) (Figure 7), which differed from that observed in **5**. The positive optical rotation exhibited by **11** {${\left[\alpha \right]}_{D}^{21}$ +12.7 (*c* 0.13, MeOH)} provided additional evidence that its flavonoid-sesquiterpenoid connection pattern was opposite to that observed in **5** {${\left[\alpha \right]}_{D}^{21}$ −195.0 (*c* 0.45, MeOH)}. The thorough examination of spectroscopic data provided unequivocal evidence for the structure of **11**, which was hence referred to as simisyzygin H4.

Compounds **12**–**14** were purified as yellowish gums, each possessing a molecular formula of C_32_H_40_O_5_. NMR analysis revealed remarkable structural similarities among these three compounds. COSY correlations between H-6ʺ/H-7ʺ and HMBC correlations from H-9ʺ to C-1ʺ/C-7ʺ/C-8ʺ/C-10ʺ established a cyclohexane ring (Figure 2). HMBC signals from H_3_-14ʺ to C-1ʺ/C-9ʺ/C-10ʺ positioned a methyl group at the sp³ hybridized C-10ʺ, while an isopropyl substituent at C-7ʺ was evidenced by COSY correlations of H-7ʺ/H-11ʺ, H-11ʺ/H-12ʺ, H-11ʺ/H-13ʺ and corresponding HMBC correlations. The cyclopentane moiety was evidenced by HMBC correlations from H-2ʺ to C-1ʺ/C-4ʺ/C-5ʺ and from H-5ʺ to C-2ʺ/C-3ʺ. COSY correlations between H-5ʺ/H-6ʺ and HMBC correlations from H-2ʺ to C-6ʺ and H-10ʺ to C-5ʺ indicated that a unique cyclopropane ring was formed at the junction between cyclohexane and cyclopentane units through C-1ʺ and C-5ʺ―C-6ʺ linkages. The remarkably upfield-shifted proton signals at *δ*_H_ 0.38 (d, *J* = 3.2 Hz, H-5ʺ) and *δ*_H_ 0.63 (d, *J* = 3.2 Hz, H-6ʺ) provided compelling evidence for this cyclopropane structure (Table 3). For compound **12**, the sesquiterpenoid moiety was characterized as a cubebene-type scaffold with a 5/3/6 ring system. This structural assignment was further supported by comparison with *β*-cubebene, which possesses a similar skeleton and closely matching spectroscopic features (Shindo et al. 2018). Finally, key HMBC correlations from H-15ʺ to C-3ʺ/C-4ʺ/C-5ʺ, from H-5ʺ to C-15ʺ, and from H-3ʺ to C-6 indicated that the cubebene-type sesquiterpenoid was connected to the A-ring of the flavonoid, forming a spiro[4.5]decane system with C-4ʺ as the spirocarbon. Compounds **13** and **14** shared the same planar structure as **12**, all belonging to spiro-meroterpenoids with their junctions at C-4ʺ. NOESY experiments revealed that **12** and **13** exhibited an sB-type spiro configuration characterized by correlations between H-15ʺa/H-3ʺ and H-15ʺb/H-5ʺ, while compound **14** displayed a sA-type configuration evidenced by H-15ʺa/H-5ʺ and H-15ʺb/H-3ʺ (Figures 3 and 4). Additionally, the relative configuration of the sesquiterpenoid portion in **12** was established through NOESY experiments, which revealed *α*-orientations for H-5ʺ, H-7ʺ, CH_3_-14ʺ, and H-15ʺb, while H-6ʺ and H-15ʺa were *β*-oriented. Structural analysis of compound **13** via NOESY correlations between H-7ʺ/H-10ʺ and H-10ʺ/H-15ʺb indicated that H-10ʺ occupied an *α*-orientation, thus placing CH_3_-14ʺ in the *β*-orientation, distinguishing **13** stereochemically from **12**. The sesquiterpenoid moiety in **14**, however, exhibited the same relative stereochemical arrangement as that of **12**. Based on these stereochemical differences, **12**–**14** were characterized as stereoisomers and designated as simisyzygins I1, I2, and I3, respectively.

Compounds **15** and **16** were isolated as yellowish gums with a molecular formula of C_32_H_40_O_5_ established by HRESIMS. Both of them were characterized as flavonoid-sesquiterpene hybrids possessing a 5-7-dioxo-6,8-dimethylflavanone skeleton analogous to the above compounds, as confirmed by comprehensive 1D and 2D NMR analyses (Table 4; Figure 2). The sesquiterpenoid portion of **15** featured a distinctive trisubstituted olefin, as evidenced by characteristic resonances at C-5ʺ (*δ*_H_ 4.92; *δ*_C_ 125.8) and C-6ʺ (*δ*_C_ 144.2). Structural connectivity was established through ^1^H–^1^H COSY correlations of H-7ʺ/H-8ʺ, H-8ʺ/H-9ʺ, H-7ʺ/H-11ʺ, H-12ʺ/H-11ʺ/H-13ʺ, and key HMBC correlations from H_3_-14ʺ to C-1ʺ/C-9ʺ/C-10ʺ, and from H-5ʺ to C-1ʺ/C-3ʺ/C-4ʺ/C-7ʺ, identifying the sesquiterpene as a 6/6 ring cadinene-type (Δ^5ʺ,6ʺ^ decalin system) with methyl and isopropyl substituents located at C-10ʺ and C-7ʺ, respectively. This sesquiterpenoid configuration was further confirmed by comparison with 1-epibicyclosesquiphellandrene, which exhibited similar spectroscopic signals (Terhune et al. 1974). Key HMBC correlations from H-5ʺ to C-6, from H_3_-11 to C-4ʺ, and from H-15ʺ to C-3ʺ/C-4ʺ/C-5ʺ established the spiro-connection between the cadinene-type sesquiterpenoid and the flavonoid at C-4ʺ. Compound **16** exhibited an identical planar structure to that of **15**. The stereochemical features of **15** and **16** were elucidated through NOESY experiments (Figure 3), which revealed that both compounds adopted an sB-type spiro configuration with characteristic correlations between H-15ʺa/H-3ʺ and H-15ʺb/H-5ʺ (Figure 4). The key stereochemical difference between these compounds was the spatial orientation of the tertiary methyl group (CH_3_-14ʺ) located at sp^3^ C-10ʺ. In **15**, NOESY correlations between H-15ʺa/H-8ʺb and H-8ʺb/H-10ʺ positioned H-10ʺ and H-15ʺa on the same side, thereby placing CH_3_-14ʺ in the opposite orientation. Conversely, in **16**, NOESY correlations between H-15ʺa/H-9ʺb and H-9ʺb/H_3_-14ʺ indicated the coplanar arrangement of CH_3_-14ʺ and H-15ʺa on the same face of the molecule. This configuration was further supported by observable chemical shift variations in their NMR data at positions C-1ʺ, C-10ʺ, and C-14ʺ. Taken together, the spectroscopic data allowed the structures of **15** and **16** to be fully determined, and they were trivially named simisyzygins J1 and J2, respectively.

Compounds **17** and **18** were purified as colorless gums sharing the identical molecular formula C_32_H_40_O_5_ determined by HRESIMS analysis. Both 1D and 2D NMR data confirmed the presence of a 5,7-dioxo-6,8-dimethylflavanone skeleton connected to a sesquiterpene via a spiro ring, as observed in **12**–**16** (Table 4; Figures 2 and 3), while significant structural differences were noted in the sesquiterpenoid portion. For **17** and **18**, analysis of HMBC and COSY correlations revealed a 6/6 ring system, similar to **15** and **16**. However, the sp^3^ quaternary nature of C-1ʺ (*δ*_C_ 41.1) was confirmed by DEPT experiments, and HMBC correlations from H_3_-14ʺ to C-1ʺ/C-2ʺ/C-6ʺ/C-10ʺ and from H-6ʺ to C-14ʺ positioned the methyl group (CH_3_-14ʺ) at C-1ʺ. Moreover, the connectivity between C-5ʺ and C-10ʺ was established through a COSY correlation between H-5ʺ/H-10ʺ and HMBC correlations from H-5ʺ to C-9ʺ/C-10ʺ and from H-10ʺ to C-6ʺ, forming an intricate tricyclic architecture (tricyclo[4.4.0.0^2,7^]decane) characteristic of a copaene-type sesquiterpenoid framework. This structural assignment was further supported by the similarity of chemical shift patterns to those of *β*-copaene (Wenkert et al. 1992). Stereochemical analysis of **17** via NOESY experiments revealed an sB-type spiro configuration, evidenced by correlations between H-15ʺa/H-3ʺb and H-15ʺb/H-5ʺ (Figures 3 and 4). The relative stereochemistry was further defined by NOESY correlations of H-5ʺ/H-7ʺ, H-5ʺ/H-10ʺ, H-5ʺ/H-15ʺb, and H-10ʺ/H-15ʺb placing H-5ʺ, H-7ʺ, H-10ʺ, and H-15ʺb on the *α*-orientation, while H-6ʺ and CH_3_-14ʺ were assigned to the *β*-orientation based on H-6ʺ/H_3_-14ʺ correlation. Compound **18** shared the same planar structure as **17**. Nevertheless, NOESY correlations between H-15ʺa/H-5ʺ and H-15ʺb/H-3ʺ established that **18** exhibited a sA-type spiro configuration (Figures 3 and 4). Further NOESY correlations established the relative configuration of the terpenoid moiety. According to the above data, the structures of **17** and **18** were elucidated and assigned as simisyzygins K1 and K2, respectively.

The absolute configurations of all new compounds were further supported by CD analysis. The experimental CD spectra of compounds **8**–**18** (see Supporting Information) displayed Cotton effect patterns consistent with those of compound **1**, whose absolute configuration was established by single-crystal X-ray diffraction. Together with the NOESY-derived relative configurations described above and comparison with structurally related analogues (**3**–**7**, **19**–**22**), these results collectively support the absolute configurations assigned to the new compounds.

Two known triterpenoids were identified through comparison of experimental and reported spectroscopic data ([*α*]D, IR, NMR, and MS), including 3*β*-hydroxytaraxast-20-en-28-oic acid (**23**) (Wang et al. 2016) and betulinic acid (**24**) (Ding et al. 2018) (Figure 1).

### Plausible biosynthetic pathway

Based on the isolated compounds and their structural features, a plausible biosynthetic pathway for the flavonoid-sesquiterpenoid hybrids has been proposed. First, the biosynthesis of the flavonoid portion presumably began with a flavanone structure, desmethoxymatteucinol (5,7-dihydroxy-6,8-dimethylflavanone), a compound known to exist in the genus *Syzygium* (Kuo et al. 2004; Memon et al. 2015; Ninh et al. 2023; Khamto et al. 2024). The flavanone underwent oxidative modifications to give carbonyl groups at C-5 and C-7 and hydroxyl groups at C-6 and C-8, establishing the 5,7-dioxo-6,8-dihydroxy-6,8-dimethylflavanone core structure. The 1,3-dione system at C-4 and C-5 could readily undergo keto-enol tautomerization, with C-10 participating to form a stabilized enolic structure (Scheme 1).

**Scheme 1.**
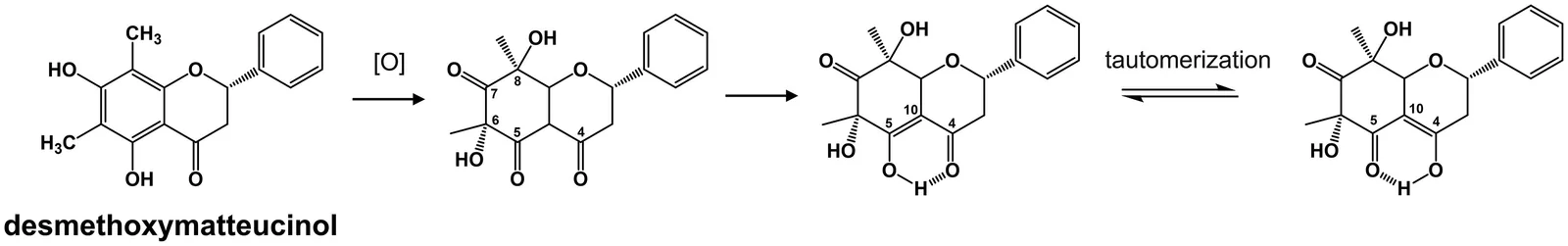
Biosynthesis of the flavonoid portion.

The formation of flavonoid-sesquiterpenoid hybrids involved different linkages between the flavonoid scaffold and various sesquiterpenoid units. The isolated compounds represented flavonoid-sesquiterpenoid hybrids that could be further categorized into five distinct structural types based on their attached flavonoid moieties: type I (**1–5**, and **8**–**18**), type II (**6**–**7**), type III (**19**–**20**), type IV (**21**), and type V (**22**). For type I compounds, the biosynthetic pathway could be divided into two primary coupling modes. The coupling mode depicted in Scheme 2 involved the connection of modified flavonoids to sesquiterpenoids (cadinene, cubebene, or copaene-types) via a spiro structure. In this process, the *π* electron of the terminal double bond at C-4ʺ in the sesquiterpenoid moiety attacked position C-6 of the flavonoid scaffold, initiating intramolecular rearrangement and dehydration to form a carbocation intermediate. Subsequently, the carbocation attacked the hydrogen at position C-9, inducing hydrogen migration and ultimately establishing a spiro connection between the sesquiterpenoid and the flavonoid. Since C-4ʺ constituted a planar chiral center capable of adopting two distinct stereochemical orientations, this process ultimately established C-4ʺ spiro junctions with either *R*-configuration of sA-type structure (**1**, **14**, and **18**) or *S*-configuration of sB-type structure (**2**, **12**–**13**, and **15**–**17**) (Figure 4). Scheme 3 illustrated the coupling mode involving ring fusion connections, in which the modified flavonoids were connected to germacrene-type sesquiterpenoids via two points (C-4ʺ and C-5ʺ) on the A-ring. Specifically, the *π* electron of the endocyclic double bond in the sesquiterpenoid moiety attacked the flavonoid at C-6. The intermediate subsequently attacked the hydrogen at C-9, generating a ring-fused bicyclo[8.4.0]tetradecane framework. Given that the ten-membered germacrene ring presented two possible stereochemical faces, attacking the flavonoid from different faces resulted in distinct connection patterns: C-4ʺ─C-6, C-5ʺ─C-9 of rA-type structure (**3**–**5**, and **8**–**9**), or C-4ʺ─C-9, C-5ʺ─C-6 of rB-type structure (**10**–**11**) (Figure 7).

**Scheme 2.**
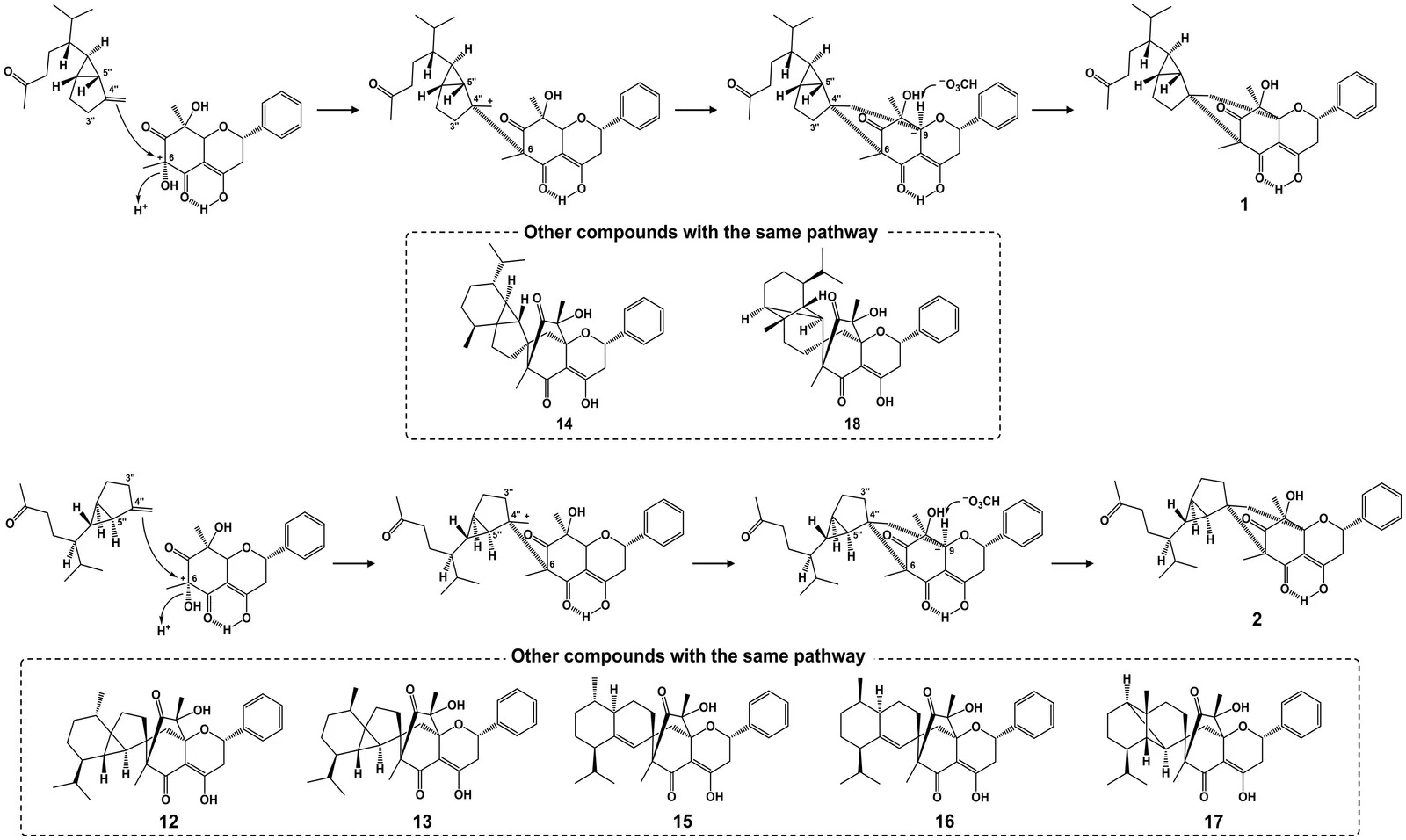
Biosynthesis of type I compounds (**1**, **2**, and **12**–**18**) through spiro connection.

**Scheme 3.**
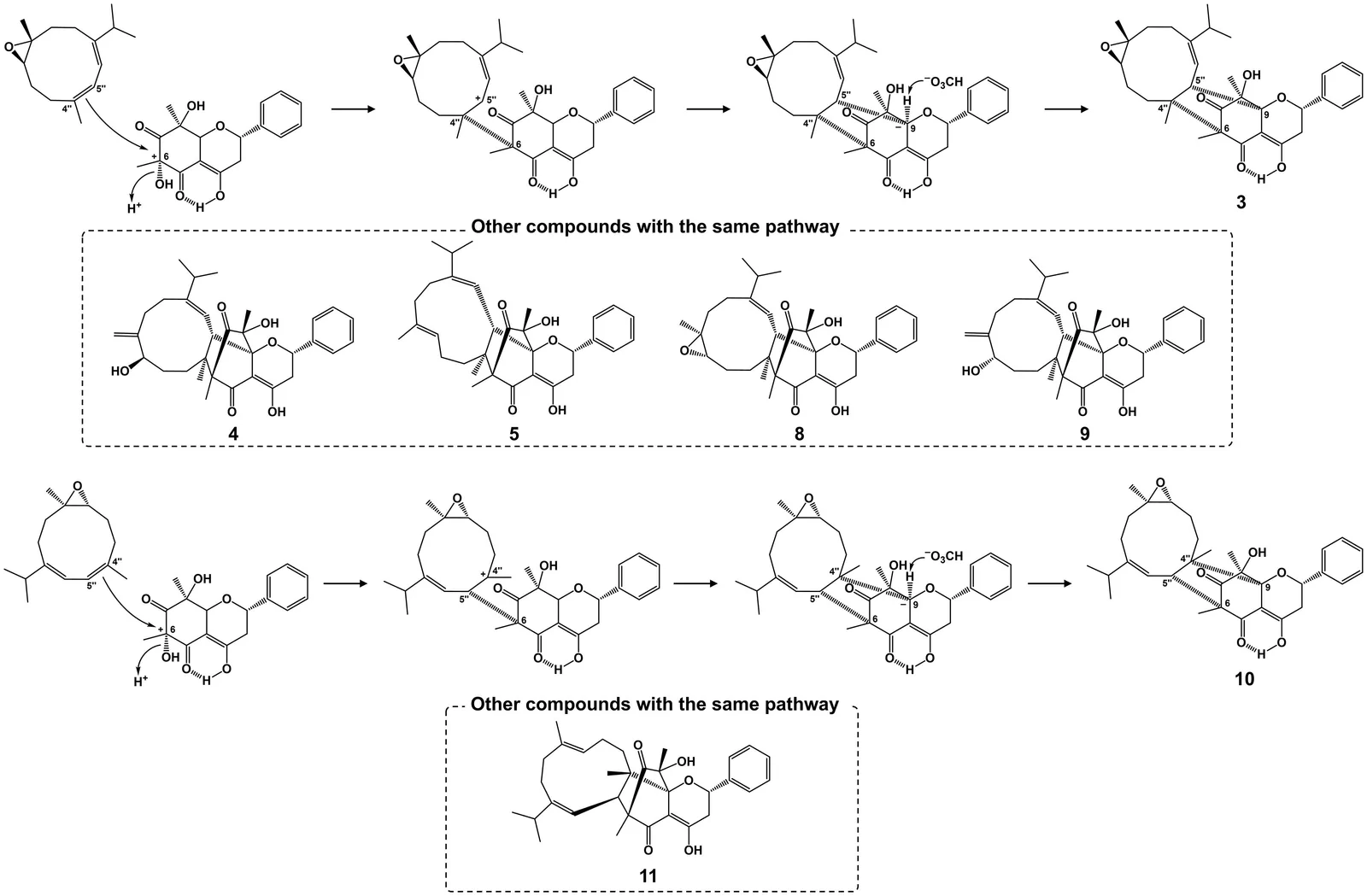
Biosynthesis of type I compounds (**3–5**, and **8**–**11**) through ring fusion connection.

Scheme 4 presented the proposed biogenesis of type II compounds. The flavonoid precursor first underwent conversion from the enol to keto form at the C-4 position, followed by an oxidation reaction, resulting in the cleavage of the C-5─C-6 bond and opening of the A-ring. Due to its *α*-keto acid character, this intermediate readily underwent decarboxylation.

**Scheme 4.**
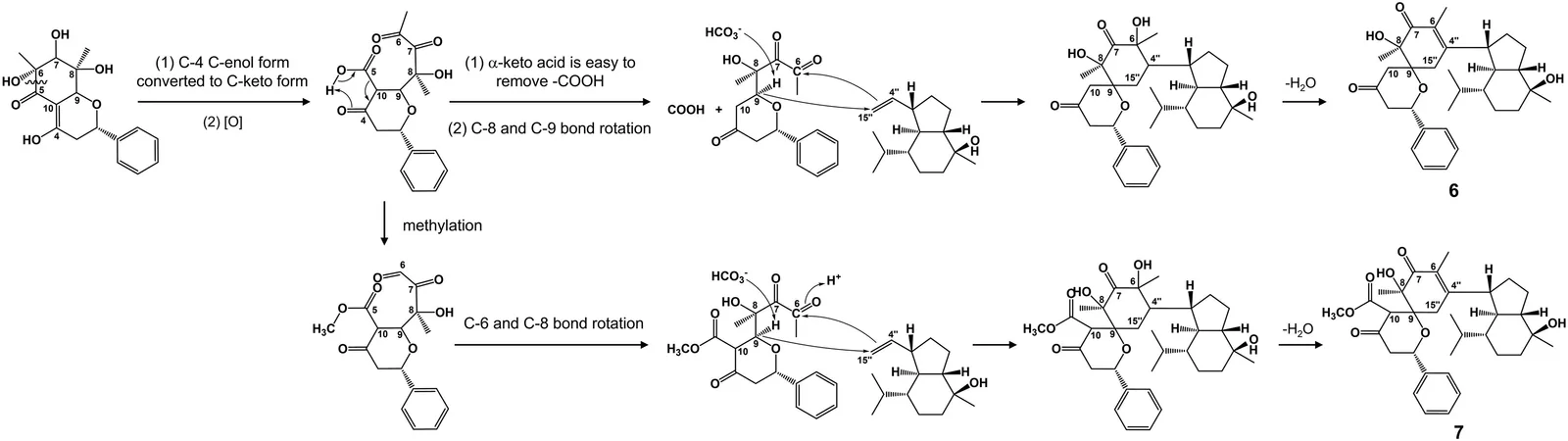
Biosynthesis of type II compounds (**6**–**7**).

The resulting intermediate then participated in a coupling sequence analogous to that observed in type I hybrids. The sesquiterpenoid component, functioning as a nucleophile through its terminal double bond π electrons, attacked the electrophilic C-6 ketone of the modified flavonoid. This attack triggered a series of intramolecular electron rearrangements and stereoelectronic effects, followed by dehydration, ultimately constructing the spiro skeletal structure of **6**. The formation of **7** followed an analogous pathway. In this pathway, the initial oxidized intermediate underwent methylation, precisely introducing a methoxy group (-OCH_3_) at the C-5 position. This methylated intermediate then proceeded through an identical coupling and rearrangement sequence as observed for compound **6**, ultimately forming compound **7** with its characteristic methoxycarbonyl substitution.

Scheme 5 illustrated the biosynthesis of type III compounds. HCO_3_^-^, as a basic reagent, selectively abstracted the C-9 proton from the intermediate, generating a highly reactive C-9 carbanion. This carbanion promptly initiated a nucleophilic attack on the pyrophosphate-modified sesquiterpenoid unit, triggering the redistribution and transfer of electrons.

**Scheme 5.**
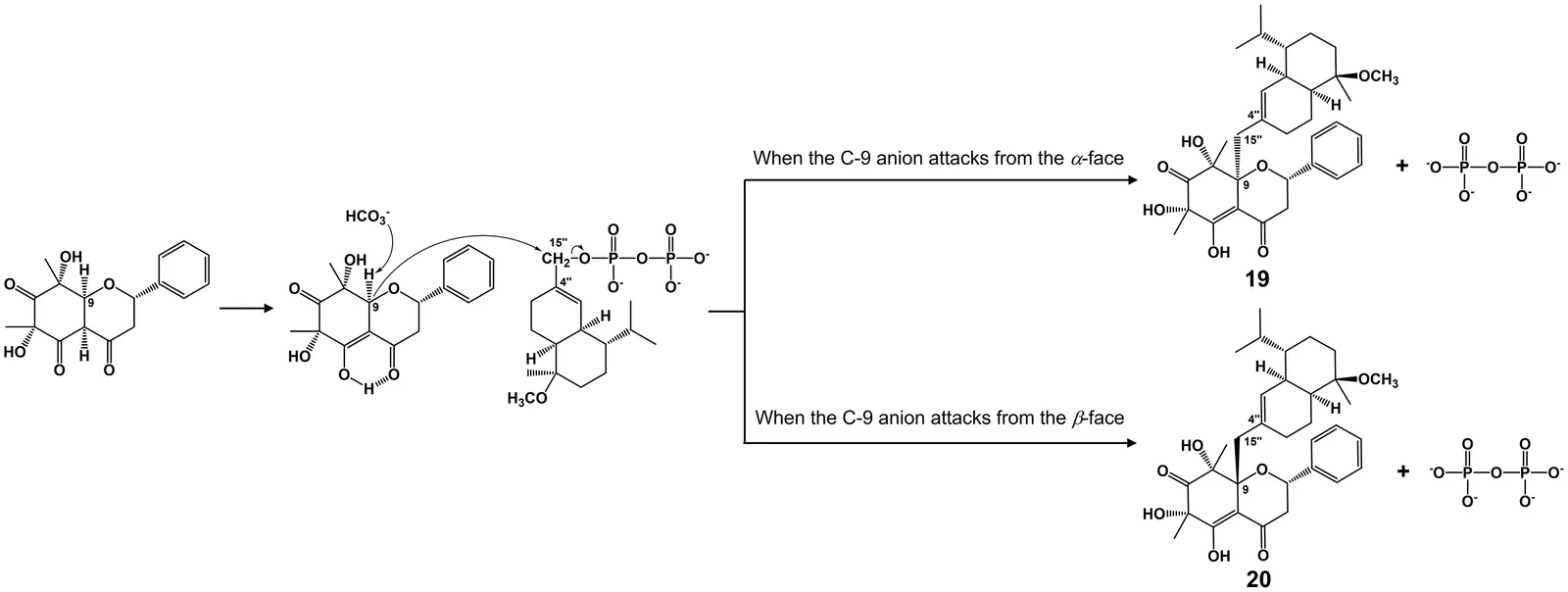
Biosynthesis of type III compounds (**19**–**20**).

The stereoselectivity of this reaction had a decisive impact on the final product structure. When the C-9 anion attacked from the *α*-face of the molecule, it stereospecifically directed the formation of **19**, accompanied by the release of pyrophosphate (PPi) as a good leaving group; conversely, when the anion attacked from the *β*-face, the reaction stereoselectively produced **20**.

The biosynthetic pathway of **21** (Scheme 6) began with an oxidation reaction of the flavonoid precursor, which resulted in the cleavage of the C-5─C-10 bond, oxidation of C-5 to a carboxyl group, and hydroxylation at C-10. Subsequently, the C-5 carboxyl group underwent lactonization with the C-8 hydroxyl group, accompanied by dehydration to form a lactone ring. A second dehydration reaction formed a C = C double bond between C-9 and C-10. The coupling reaction mechanism resembled the biosynthesis of type I hybrids, that the *π* electrons from the terminal double bond attacked the endocyclic double bond (C-9) of the intermediate, forming new carbon-carbon bonds, and generating a bridged structure. Finally, through tautomerization, the molecule rearranged to its thermodynamically favored configuration to form **21**. While the synthetic pathway of **22** was similar, the difference lay in the formation of the starting precursor. The flavonoid precursor underwent an oxidation reaction resulting in the cleavage of bonds between C-5/C-6 and C-8/C-9, with the oxidation at C-9 to a carboxyl group, and the hydroxylation at C-10. These structural differences determined the final structure of **22**.

**Scheme 6.**
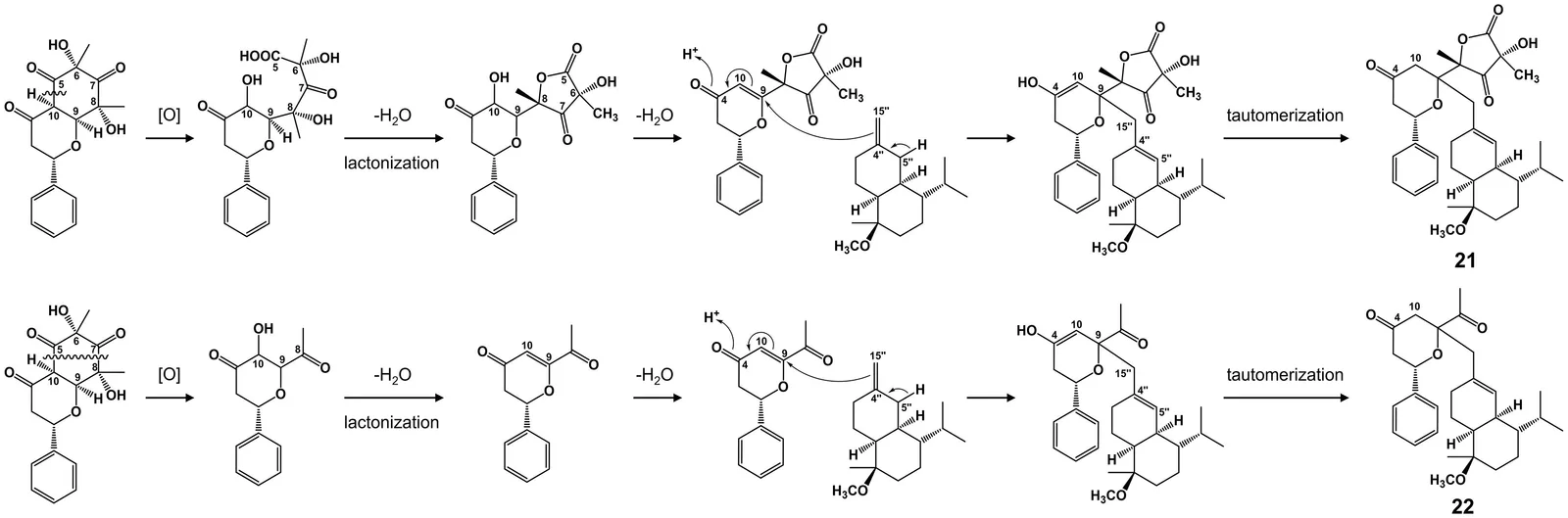
Biosynthesis of type IV and V compounds (**21** and **22**, respectively).

These proposed biosynthetic sequences not only explained the diverse connection patterns between flavonoid and sesquiterpenoid moieties, but also revealed the specialized molecular mechanisms by which *Syzygium* species produce these unique hybrid metabolites.

### Biological activity assessment

#### Anti-inflammatory activity assessment

Except for some compounds available in insufficient amounts for testing, this study evaluated the activities of 12 compounds (compounds **1**–**8**, **11**–**12**, **16**, and **19**) to develop new anti-inflammatory agents. Anti-inflammatory activity was evaluated using a human neutrophil model stimulated with *N*-formyl-methionine-leucyl-phenylalanine (fMLP)/cytochalasin B (CB). In this model, the bacterial peptide fMLP stimulated human neutrophils, with CB enhancing the inflammatory response, causing superoxide anion generation and elastase release from neutrophils. The inhibitory effects of test compounds on these two inflammatory mediators could then be assessed.

The experimental results are shown in Table 5. Among the tested compounds, except for **6** and **7**, which showed moderate activities, the other hybrids with similar structural scaffolds exhibited excellent inhibitory effects on both inflammatory mediators. These active compounds displayed IC_50_ values ranging from 0.04 ± 0.02 to 1.78 ± 0.27 μM for the inhibition of superoxide anion generation, and from 0.16 ± 0.03 to 2.04 ± 0.59 μM for the inhibition of elastase release. Notably, most compounds had IC_50_ values below 1 μM, exceeding the potency of the positive control LY294002, with inhibition percentages generally exceeding 100% and showing statistically significantly up to 32-fold greater compared to the control group (*p* < 0.001), demonstrating their exceptional potential. Particularly, **3** exhibited the most prominent activity, with IC_50_ values of 0.04 ± 0.02 μM and 0.16 ± 0.03 μM for superoxide anion generation and elastase release, respectively, achieving nanomolar-level potency without exhibiting noticeable cytotoxicity.

**Table 5.** Inhibitory effects of isolates on superoxide anion generation and elastase release in fMLP/CB-induced human neutrophils.

Compound	Superoxide anion		Elastase release	
	IC_50_ (µM)	Inh%	IC_50_ (µM)	Inh%
simisyzygin A (**1**)	0.39 ± 0.14	102.02 ± 0.90***	0.76 ± 0.22	120.13 ± 6.41***
simisyzygin B (**2**)	0.83 ± 0.18	102.53 ± 0.48***	2.04 ± 0.59	109.09 ± 6.84***
simisyzygin C (**3**)	0.04 ± 0.02	100.17 ± 0.46***	0.16 ± 0.03	109.63 ± 4.05***
simisyzygin D (**4**)	0.18 ± 0.01	100.05 ± 0.42***	0.23 ± 0.02	100.32 ± 2.13***
simisyzygin E (**5**)	0.15 ± 0.02	104.05 ± 0.48***	0.35 ± 0.10	116.41 ± 3.51***
simisyzygin F (**6**)	> 10	33.73 ± 1.21***	> 10	47.64 ± 3.96***
simisyzygin G (**7**)	4.38 ± 0.37	86.26 ± 3.70***	4.44 ± 0.26	74.28 ± 1.64***
simisyzygin H1 (**8**)	0.14 ± 0.02	100.54 ± 0.93***	0.32 ± 0.02	107.72 ± 1.80***
simisyzygin H4 (**11**)	NT	NT	0.40 ± 0.06	109.19 ± 0.94***
simisyzygin I1 (**12**)	0.61 ± 0.17	86.60 ± 6.14*** (at 3 μM)	0.59 ± 0.10	117.33 ± 2.10***
simisyzygin J2 (**16**)	1.78 ± 0.27	86.83 ± 5.36***	1.21 ± 0.11	109.59 ± 1.12***
simicadinene A (**19**)	0.59 ± 0.12	100.87 ± 1.34***	1.16 ± 0.24	112.79 ± 2.78***
^#^LY294002	1.29 ± 0.52	–	2.61 ± 0.72	–

Percentage of inhibition (Inh%) at 10 µM. Results are presented as mean ± S.E.M. (*n* = 3–4).

*p < 0.05,

**p < 0.01,

***p < 0.001 compared with the control (DMSO).

^#^LY294002, an inhibitor of phosphoinositide 3-kinase (PI3K), was used as a positive control in anti-inflammatory assays. NT = non test.

#### Structure-activity relationship (SAR)

The SAR of flavonoid-sesquiterpenoid compounds was analyzed according to their inhibitory effects on superoxide anion generation and elastase release (Figure 8). Among the flavonoid portion in Frame A, type I flavanones (**1**–**5**, **8**, **11**–**12**, and **16**), characterized by modified tetracyclic structures, exhibited the most potent anti-inflammatory effects with IC_50_ values generally below 1 μM and represented the most prevalent scaffold in this series. Type III compound (**19**), comprising tricyclic flavanones, displayed moderate inhibitory activity against inflammatory mediators. Type II compounds (**6**–**7**), featuring tricyclic flavanones with spiro configuration, demonstrated the lowest potency among the evaluated structures. Comparative analysis between **6** (IC_50_ >10 μM for superoxide anion generation; IC_50_ >10 μM for elastase release) and **7** (IC_50_ = 4.38 μM for superoxide anion generation; IC_50_ = 4.44 μM for elastase release) revealed that methoxycarbonyl group at the C-5 position significantly enhanced the anti-inflammatory activity, suggesting the importance of this position for optimal biological response.

**Figure 8.**
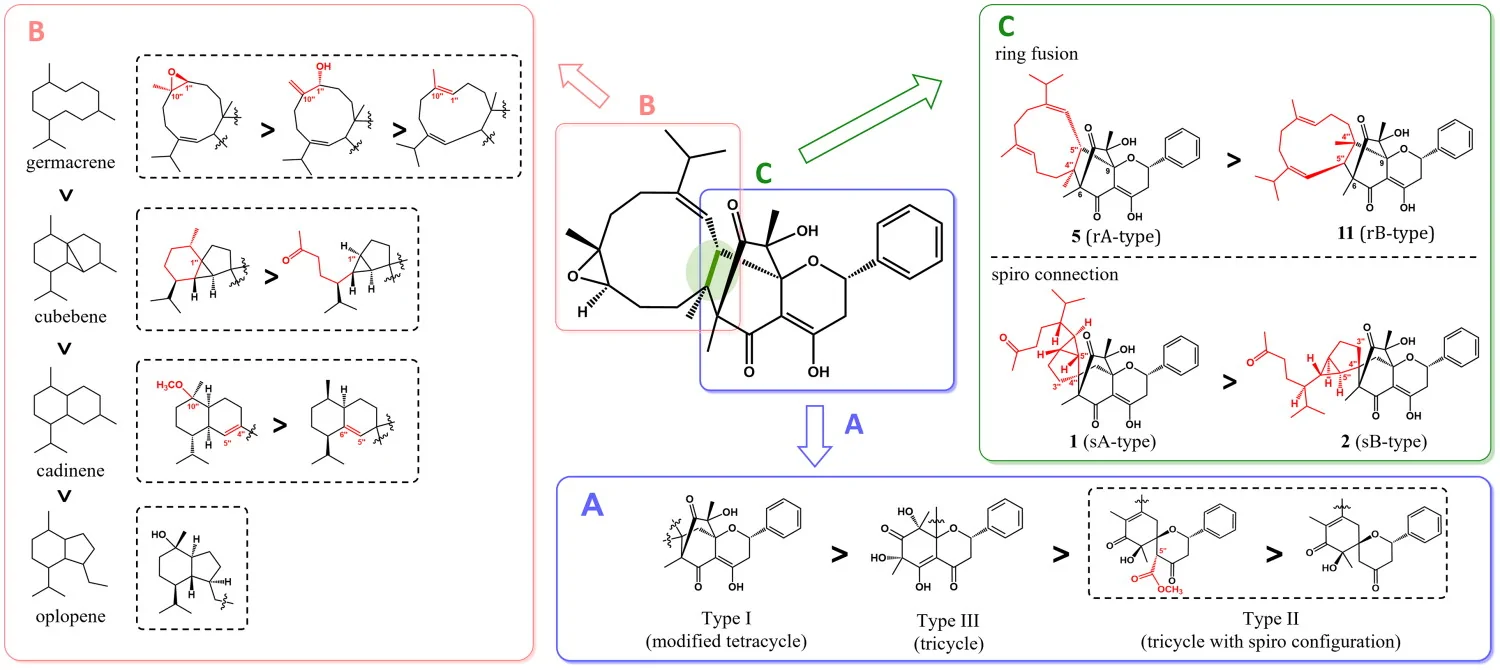
SAR of the results for anti-inflammatory activities.

For the sesquiterpenoid moieties in Frame B, a distinct activity hierarchy was observed in the order: germacrene-type (**3**–**5**, **8**, and **11**) > cubebene-type (**1**–**2**, and **12**) > cadinene-type (**16** and **19**) > oplopene-type (**6**–**7**). Within the germacrene-type derivatives, the incorporation of an epoxide group between C-1ʺ and C-10ʺ positions (**3** and **8**, IC_50_ = 0.04 and 0.14 μM for superoxide anion generation; IC_50_ = 0.16 and 0.32 μM for elastase release) conferred the best potency against both inflammatory mediators. The cubebene-type scaffold with a complete cyclohexane structure (**12**, IC_50_ = 0.61 μM for superoxide anion generation; IC_50_ = 0.59 μM for elastase release) demonstrated superior activity compared to its ring-opened analog (*seco*-cubebene-type) (**1** and **2**, IC_50_ = 0.39 and 0.83 μM for superoxide anion generation; IC_50_ = 0.76 and 2.04 μM for elastase release). For cadinene-type structures, Δ^4ʺ,5ʺ^ decalin with a methoxy substitution at C-10ʺ (**19**, IC_50_ = 0.59 μM for superoxide anion generation; IC_50_ = 1.16 μM for elastase release) exhibited enhanced anti-inflammatory properties compared to the Δ^5ʺ,6ʺ^ decalin configuration (**16**, IC_50_ = 1.78 μM for superoxide anion generation; IC_50_ = 1.21 μM for elastase release).

Regarding the connection mode between flavonoid and sesquiterpenoid moieties in Frame C, two distinct linking patterns were identified: ring fusion and spiro connection. Compounds featuring ring fusion patterns (**3**–**5**, **8**, and **11**), where flavonoids and sesquiterpenes—predominantly of the germacrene-type—were linked through the C-4ʺ and C-5ʺ positions, showed superior anti-inflammatory properties. In contrast, compounds with spiro connection patterns (**1**–**2**, **12**, and **16**) linking flavonoids to various sesquiterpenoid types at the C-4ʺ position, showed comparatively reduced activity.

Further structural refinement revealed that within the ring fusion category, **5** and **11** shared identical flavonoid and germacrene-type sesquiterpenoid moieties, differing only in their connection patterns. The rA-type (**5**, IC_50_ = 0.15 μM for superoxide anion generation; IC_50_ = 0.35 μM for elastase release) exhibited higher potency than the rB-type (**11**, IC_50_ = 0.40 μM for elastase release). Similarly, **1** and **2** with spiro connection patterns shared identical flavonoid and *seco*-cubebene sesquiterpenoid components. The sA-type (**1**, IC_50_ = 0.39 μM for superoxide anion generation; IC_50_ = 0.76 μM for elastase release) demonstrated enhanced efficacy compared to the sB-type analog (**2**, IC_50_ = 0.83 μM for superoxide anion generation; IC_50_ = 2.04 μM for elastase release). These findings provide valuable guidance for developing more effective anti-inflammatory compounds in this series.

## Conclusions

In total, our investigation of *S. simile* leaves has yielded 22 flavonoid-sesquiterpenoid hybrids (**1**–**22**) and two known triterpenoids (**23**–**24**). These 22 hybrids comprised five flavonoid scaffolds (I–V) and five sesquiterpenoid types (germacrene-, cubebene-, oplopene-, cadinene-, and copaene-type), linked via ring fusion, spiro junctions, or single-bond connections. Notably, compounds with similar skeletal frameworks have been discovered in *S. oblanceolatum* (Koga et al. 2024a; 2024b), suggesting that these unique molecular scaffolds may serve as chemotaxonomic markers for the genus *Syzygium*, providing important evidence for chemotaxonomic studies of this plant genus.

The proposed biosynthetic pathway for these hybrids likely initiated with the oxidative transformation of desmethoxymatteucinol to generate a key flavonoid core structure, which subsequently underwent a series of enzymatic reactions to form different types of flavonoid scaffolds. The final biosynthesis involved electron transfer from the exocyclic methylene moiety of the cyclic sesquiterpenoids, facilitating nucleophilic attack on the flavonoid skeleton to form these distinctive hybrid natural product architectures.

Evaluation of the anti-inflammatory properties of this compound series revealed exceptional inhibitory effects against superoxide anion generation and elastase release, with most compounds showing IC_50_ values up to 32-fold lower than that of positive control, without significant cytotoxicity at the tested concentration. Detailed SAR analysis elucidated the correlation between molecular structural features and anti-inflammatory activity, identifying simisyzygin C (**3**) as the most potent anti-inflammatory agent, characterized by a type I flavonoid connected to a germacrene-type sesquiterpenoid through an rA-type ring fusion pattern, with IC_50_ values reaching the nanomolar range. These SAR findings not only revealed the key structural factors influencing biological activity but also provided clear directions for further structural optimization. The exceptional anti-inflammatory properties and favorable safety profiles exhibited by these structurally unique flavonoid-sesquiterpenoid hybrids make them highly promising candidates warranting further development as effective therapeutic agents for inflammatory diseases.

## Supplementary Material

Supplementary Materials.docx

## Data Availability

The data presented in this study are available in Supplementary Materials.
