# Supplementary material for: Discovery of potent anti-inflammatory flavonoid-sesquiterpenoid hybrids from Syzygium simile: Structure–activity relationships and nanomolar inhibition of human neutrophil activation
Source: Pharm Biol. 2026 Oct 28;64(1):1143–67. doi: 10.1080/13880209.2026.2746239 (PMC13717781; doi:10.1080/13880209.2026.2746239)
Supplement: Supplementary file 1 [file IPHB_A_2746239_SM6224.docx]

**Supporting Information**

Discovery of Potent Anti-inflammatory Flavonoid-Sesquiterpenoid Hybrids from *Syzygium simile*: Structure–Activity Relationships and Nanomolar Inhibition of Human Neutrophil Activation

Yu-Chun Lin^a^, Ho-Cheng Wu^a.b^, Tsong-Long Hwang^c^, Yueh-Hsiung Kuo^d,e^, Chu-Hung Lin^f^, Horng-Huey Ko^a,b,g,h^, Hsun-Shuo Chang^a,b,g,*^

*^a^*School of Pharmacy, Kaohsiung Medical University, Kaohsiung 807, Taiwan.

*^b^*Department of Medical Research, Kaohsiung Medical University Hospital, Kaohsiung 807, Taiwan.

*^c^*Graduate Institute of Healthy Industry Technology, Chang Gung University of Science and Technology, Taoyuan 333, Taiwan.

*^d^*Department of Chinese Pharmaceutical Sciences and Chinese Medicine Resources, Chinese Medicine Research Center, China Medical University, Taichung 404, Taiwan.

*^e^*Department of Biotechnology, Asia University, Taichung 413, Taiwan.

*^f^*Biomedical Technology and Device Research Laboratories, Industrial Technology Research Institute, Hsinchu 310, Taiwan.

*^g^*Drug Development and Value Creation Research Center, Kaohsiung Medical University, Kaohsiung 807, Taiwan.

*^h^*Department of Pharmacy, Kaohsiung Municipal Siaogang Hospital, Kaohsiung 812, Taiwan.

*Corresponding author at: School of Pharmacy, Kaohsiung Medical University, Kaohsiung 807, Taiwan; Orcid.org/0000-0003-2745-139X; Phone: +886-7-3121101 ext. 2664; Email: hschang@kmu.edu.tw; Fax: +886-7-3210683 (H.-S. Chang).

*Keywords*: *Syzygium simile*, flavonoid-sesquiterpenoid hybrids, anti-inflammatory activity

[Figure S1. Simisyzygin A (1) CD spectrum (MeOH)	5](#_Toc239856960)

[Figure S2. Simisyzygin A (1) HRESIMS spectrum	5](#_Toc239856961)

[Figure S3. Simisyzygin A (1) ^1^H NMR spectrum (CDCl_3_, 400 MHz)	6](#_Toc239856962)

[Figure S4. Simisyzygin A (1) ^13^C NMR spectrum (CDCl_3_, 100 MHz)	6](#_Toc239856963)

[Figure S5. Simisyzygin A (1) DEPT spectrum	7](#_Toc239856964)

[Figure S6. Simisyzygin A (1) HSQC spectrum	7](#_Toc239856965)

[Figure S7. Simisyzygin A (1) COSY spectrum	8](#_Toc239856966)

[Figure S8. Simisyzygin A (1) HMBC spectrum	8](#_Toc239856967)

[Figure S9. Simisyzygin A (1) NOESY spectrum	9](#_Toc239856968)

[Figure S10. Simisyzygin B (2) CD spectrum (MeOH)	9](#_Toc239856969)

[Figure S11. Simisyzygin B (2) HRESIMS spectrum	10](#_Toc239856970)

[Figure S12. Simisyzygin B (2) ^1^H NMR spectrum (CDCl_3_, 400 MHz)	10](#_Toc239856971)

[Figure S13. Simisyzygin B (2) ^13^C NMR spectrum (CDCl_3_, 100 MHz)	11](#_Toc239856972)

[Figure S14. Simisyzygin B (2) DEPT spectrum	11](#_Toc239856973)

[Figure S15. Simisyzygin B (2) HSQC spectrum	12](#_Toc239856974)

[Figure S16. Simisyzygin B (2) COSY spectrum	12](#_Toc239856975)

[Figure S17. Simisyzygin B (2) HMBC spectrum	13](#_Toc239856976)

[Figure S18. Simisyzygin B (2) NOESY spectrum	13](#_Toc239856977)

[Figure S19. Simisyzygin H1 (8) CD spectrum (MeOH)	14](#_Toc239856978)

[Figure S20. Simisyzygin H1 (8) HRESIMS spectrum	14](#_Toc239856979)

[Figure S21. Simisyzygin H1 (8) ^1^H NMR spectrum (CDCl_3_, 400 MHz)	15](#_Toc239856980)

[Figure S22. Simisyzygin H1 (8) ^13^C NMR spectrum (CDCl_3_, 100 MHz)	15](#_Toc239856981)

[Figure S23. Simisyzygin H1 (8) DEPT spectrum	16](#_Toc239856982)

[Figure S24. Simisyzygin H1 (8) HSQC spectrum	16](#_Toc239856983)

[Figure S25. Simisyzygin H1 (8) COSY spectrum	17](#_Toc239856984)

[Figure S26. Simisyzygin H1 (8) HMBC spectrum	17](#_Toc239856985)

[Figure S27. Simisyzygin H1 (8) NOESY spectrum	18](#_Toc239856986)

[Figure S28. Simisyzygin H2 (9) CD spectrum (MeOH)	18](#_Toc239856987)

[Figure S29. Simisyzygin H2 (9) HRESIMS spectrum	19](#_Toc239856988)

[Figure S30. Simisyzygin H2 (9) ^1^H NMR spectrum (CDCl_3_, 600 MHz)	19](#_Toc239856989)

[Figure S31. Simisyzygin H2 (9) ^13^C NMR spectrum (CDCl_3_, 150 MHz)	20](#_Toc239856990)

[Figure S32. Simisyzygin H2 (9) DEPT spectrum	20](#_Toc239856991)

[Figure S33. Simisyzygin H2 (9) HSQC spectrum	21](#_Toc239856992)

[Figure S34. Simisyzygin H2 (9) COSY spectrum	21](#_Toc239856993)

[Figure S35. Simisyzygin H2 (9) HMBC spectrum	22](#_Toc239856994)

[Figure S36. Simisyzygin H2 (9) NOESY spectrum	22](#_Toc239856995)

[Figure S37. Simisyzygin H3 (10) CD spectrum (MeOH)	23](#_Toc239856996)

[Figure S38. Simisyzygin H3 (10) HRESIMS spectrum	23](#_Toc239856997)

[Figure S39. Simisyzygin H3 (10) ^1^H NMR spectrum (CDCl_3_, 600 MHz)	24](#_Toc239856998)

[Figure S40. Simisyzygin H3 (10) ^13^C NMR spectrum (CDCl_3_, 150 MHz)	24](#_Toc239856999)

[Figure S41. Simisyzygin H3 (10) DEPT spectrum	25](#_Toc239857000)

[Figure S42. Simisyzygin H3 (10) HSQC spectrum	25](#_Toc239857001)

[Figure S43. Simisyzygin H3 (10) COSY spectrum	26](#_Toc239857002)

[Figure S44. Simisyzygin H3 (10) HMBC spectrum	26](#_Toc239857003)

[Figure S45. Simisyzygin H3 (10) NOESY spectrum	27](#_Toc239857004)

[Figure S46. Simisyzygin H4 (11) CD spectrum (MeOH)	27](#_Toc239857005)

[Figure S47. Simisyzygin H4 (11) HRESIMS spectrum	28](#_Toc239857006)

[Figure S48. Simisyzygin H4 (11) ^1^H NMR spectrum (CDCl_3_, 600 MHz)	28](#_Toc239857007)

[Figure S49. Simisyzygin H4 (11) ^13^C NMR spectrum (CDCl_3_, 150 MHz)	29](#_Toc239857008)

[Figure S50. Simisyzygin H4 (11) DEPT spectrum	29](#_Toc239857009)

[Figure S51. Simisyzygin H4 (11) HSQC spectrum	30](#_Toc239857010)

[Figure S52. Simisyzygin H4 (11) COSY spectrum	30](#_Toc239857011)

[Figure S53. Simisyzygin H4 (11) HMBC spectrum	31](#_Toc239857012)

[Figure S54. Simisyzygin H4 (11) NOESY spectrum	31](#_Toc239857013)

[Figure S55. Simisyzygin I1 (12) CD spectrum (MeOH)	32](#_Toc239857014)

[Figure S56. Simisyzygin I1 (12) HRESIMS spectrum	32](#_Toc239857015)

[Figure S57. Simisyzygin I1 (12) ^1^H NMR spectrum (CDCl_3_, 400 MHz)	33](#_Toc239857016)

[Figure S58. Simisyzygin I1 (12) ^13^C NMR spectrum (CDCl_3_, 100 MHz)	33](#_Toc239857017)

[Figure S59. Simisyzygin I1 (12) DEPT spectrum	34](#_Toc239857018)

[Figure S60. Simisyzygin I1 (12) HSQC spectrum	34](#_Toc239857019)

[Figure S61. Simisyzygin I1 (12) COSY spectrum	35](#_Toc239857020)

[Figure S62. Simisyzygin I1 (12) HMBC spectrum	35](#_Toc239857021)

[Figure S63. Simisyzygin I1 (12) NOESY spectrum	36](#_Toc239857022)

[Figure S64. Simisyzygin I2 (13) CD spectrum (MeOH)	36](#_Toc239857023)

[Figure S65. Simisyzygin I2 (13) HRESIMS spectrum	37](#_Toc239857024)

[Figure S66. Simisyzygin I2 (13) ^1^H NMR spectrum (CDCl_3_, 400 MHz)	37](#_Toc239857025)

[Figure S67. Simisyzygin I2 (13) ^13^C NMR spectrum (CDCl_3_, 100 MHz)	38](#_Toc239857026)

[Figure S68. Simisyzygin I2 (13) DEPT spectrum	38](#_Toc239857027)

[Figure S69. Simisyzygin I2 (13) HSQC spectrum	39](#_Toc239857028)

[Figure S70. Simisyzygin I2 (13) COSY spectrum	39](#_Toc239857029)

[Figure S71. Simisyzygin I2 (13) HMBC spectrum	40](#_Toc239857030)

[Figure S72. Simisyzygin I2 (13) NOESY spectrum	40](#_Toc239857031)

[Figure S73. Simisyzygin I3 (14) CD spectrum (MeOH)	41](#_Toc239857032)

[Figure S74. Simisyzygin I3 (14) HRESIMS spectrum	41](#_Toc239857033)

[Figure S75. Simisyzygin I3 (14) ^1^H NMR spectrum (CDCl_3_, 600 MHz)	42](#_Toc239857034)

[Figure S76. Simisyzygin I3 (14) ^13^C NMR spectrum (CDCl_3_, 150 MHz)	42](#_Toc239857035)

[Figure S77. Simisyzygin I3 (14) DEPT spectrum	43](#_Toc239857036)

[Figure S78. Simisyzygin I3 (14) HSQC spectrum	43](#_Toc239857037)

[Figure S79. Simisyzygin I3 (14) COSY spectrum	44](#_Toc239857038)

[Figure S80. Simisyzygin I3 (14) HMBC spectrum	44](#_Toc239857039)

[Figure S81. Simisyzygin I3 (14) NOESY spectrum	45](#_Toc239857040)

[Figure S82. Simisyzygin J1 (15) CD spectrum (MeOH)	45](#_Toc239857041)

[Figure S83. Simisyzygin J1 (15) HRESIMS spectrum	46](#_Toc239857042)

[Figure S84. Simisyzygin J1 (15) ^1^H NMR spectrum (CDCl_3_, 600 MHz)	46](#_Toc239857043)

[Figure S85. Simisyzygin J1 (15) ^13^C NMR spectrum (CDCl_3_, 150 MHz)	47](#_Toc239857044)

[Figure S86. Simisyzygin J1 (15) DEPT spectrum	47](#_Toc239857045)

[Figure S87. Simisyzygin J1 (15) HSQC spectrum	48](#_Toc239857046)

[Figure S88. Simisyzygin J1 (15) COSY spectrum	48](#_Toc239857047)

[Figure S89. Simisyzygin J1 (15) HMBC spectrum	49](#_Toc239857048)

[Figure S90. Simisyzygin J1 (15) NOESY spectrum	49](#_Toc239857049)

[Figure S91. Simisyzygin J2 (16) CD spectrum (MeOH)	50](#_Toc239857050)

[Figure S92. Simisyzygin J2 (16) HRESIMS spectrum	50](#_Toc239857051)

[Figure S93. Simisyzygin J2 (16) ^1^H NMR spectrum (CDCl_3_, 600 MHz)	51](#_Toc239857052)

[Figure S94. Simisyzygin J2 (16) ^13^C NMR spectrum (CDCl_3_, 150 MHz)	51](#_Toc239857053)

[Figure S95. Simisyzygin J2 (16) DEPT spectrum	52](#_Toc239857054)

[Figure S96. Simisyzygin J2 (16) HSQC spectrum	52](#_Toc239857055)

[Figure S97. Simisyzygin J2 (16) COSY spectrum	53](#_Toc239857056)

[Figure S98. Simisyzygin J2 (16) HMBC spectrum	53](#_Toc239857057)

[Figure S99. Simisyzygin J2 (16) NOESY spectrum	54](#_Toc239857058)

[Figure S100. Simisyzygin K1 (17) CD spectrum (MeOH)	54](#_Toc239857059)

[Figure S101. Simisyzygin K1 (17) HRESIMS spectrum	55](#_Toc239857060)

[Figure S102. Simisyzygin K1 (17) ^1^H NMR spectrum (CDCl_3_, 600 MHz)	55](#_Toc239857061)

[Figure S103. Simisyzygin K1 (17) ^13^C NMR spectrum (CDCl_3_, 150 MHz)	56](#_Toc239857062)

[Figure S104. Simisyzygin K1 (17) DEPT spectrum	56](#_Toc239857063)

[Figure S105. Simisyzygin K1 (17) HSQC spectrum	57](#_Toc239857064)

[Figure S106. Simisyzygin K1 (17) COSY spectrum	57](#_Toc239857065)

[Figure S107. Simisyzygin K1 (17) HMBC spectrum	58](#_Toc239857066)

[Figure S108. Simisyzygin K1 (17) NOESY spectrum	58](#_Toc239857067)

[Figure S109. Simisyzygin K2 (18) CD spectrum (MeOH)	59](#_Toc239857068)

[Figure S110. Simisyzygin K2 (18) HRESIMS spectrum	59](#_Toc239857069)

[Figure S111. Simisyzygin K2 (18) ^1^H NMR spectrum (CDCl_3_, 600 MHz)	60](#_Toc239857070)

[Figure S112. Simisyzygin K2 (18) ^13^C NMR spectrum (CDCl_3_, 150 MHz)	60](#_Toc239857071)

[Figure S113. Simisyzygin K2 (18) DEPT spectrum	61](#_Toc239857072)

[Figure S114. Simisyzygin K2 (18) HSQC spectrum	61](#_Toc239857073)

[Figure S115. Simisyzygin K2 (18) COSY spectrum	62](#_Toc239857074)

[Figure S116. Simisyzygin K2 (18) HMBC spectrum	62](#_Toc239857075)

[Figure S117. Simisyzygin K2 (18) NOESY spectrum	63](#_Toc239857076)

[Figure S118. Superoxide anion generation of different concentrations of compounds 1-8, 12, 16, 19 and LY294002	64](#_Toc239857077)

[Figure S108. Elastase release of different concentrations of compounds 1-8, 12, 16, 19 and LY294002	65](#_Toc239857078)

[**Figure S109.** HPLC purity analysis of compound **3** (peak #3, 95.047% purity).	66](#_Toc239857079)

X-ray diffraction data for compound **1** …………………….……………….……………….…………..67

Figure S1. Simisyzygin A (1) CD spectrum (MeOH)

Figure S2. Simisyzygin A (1) HRESIMS spectrum

Figure S3. Simisyzygin A (1) ^1^H NMR spectrum (CDCl_3_, 400 MHz)

Figure S4. Simisyzygin A (1) ^13^C NMR spectrum (CDCl_3_, 100 MHz)

DEPT-90

DEPT-135

Figure S5. Simisyzygin A (1) DEPT spectrum

Figure S6. Simisyzygin A (1) HSQC spectrum

Figure S7. Simisyzygin A (1) COSY spectrum

Figure S8. Simisyzygin A (1) HMBC spectrum

Figure S9. Simisyzygin A (1) NOESY spectrum

Figure S10. Simisyzygin B (2) CD spectrum (MeOH)

Figure S11. Simisyzygin B (2) HRESIMS spectrum

Figure S12. Simisyzygin B (2) ^1^H NMR spectrum (CDCl_3_, 400 MHz)

Figure S13. Simisyzygin B (2) ^13^C NMR spectrum (CDCl_3_, 100 MHz)

DEPT-90

DEPT-135

Figure S14. Simisyzygin B (2) DEPT spectrum

Figure S15. Simisyzygin B (2) HSQC spectrum

Figure S16. Simisyzygin B (2) COSY spectrum

Figure S17. Simisyzygin B (2) HMBC spectrum

Figure S18. Simisyzygin B (2) NOESY spectrum

Figure S19. Simisyzygin H1 (8) CD spectrum (MeOH)

Figure S20. Simisyzygin H1 (8) HRESIMS spectrum

Figure S21. Simisyzygin H1 (8) ^1^H NMR spectrum (CDCl_3_, 400 MHz)

Figure S22. Simisyzygin H1 (8) ^13^C NMR spectrum (CDCl_3_, 100 MHz)

DEPT-90

DEPT-135

Figure S23. Simisyzygin H1 (8) DEPT spectrum

Figure S24. Simisyzygin H1 (8) HSQC spectrum

Figure S25. Simisyzygin H1 (8) COSY spectrum

Figure S26. Simisyzygin H1 (8) HMBC spectrum

Figure S27. Simisyzygin H1 (8) NOESY spectrum

Figure S28. Simisyzygin H2 (9) CD spectrum (MeOH)

Figure S29. Simisyzygin H2 (9) HRESIMS spectrum

Figure S30. Simisyzygin H2 (9) ^1^H NMR spectrum (CDCl_3_, 600 MHz)

Figure S31. Simisyzygin H2 (9) ^13^C NMR spectrum (CDCl_3_, 150 MHz)

DEPT-135

DEPT-90

Figure S32. Simisyzygin H2 (9) DEPT spectrum

Figure S33. Simisyzygin H2 (9) HSQC spectrum

Figure S34. Simisyzygin H2 (9) COSY spectrum

Figure S35. Simisyzygin H2 (9) HMBC spectrum

Figure S36. Simisyzygin H2 (9) NOESY spectrum

Figure S37. Simisyzygin H3 (10) CD spectrum (MeOH)

Figure S38. Simisyzygin H3 (10) HRESIMS spectrum

Figure S39. Simisyzygin H3 (10) ^1^H NMR spectrum (CDCl_3_, 600 MHz)

Figure S40. Simisyzygin H3 (10) ^13^C NMR spectrum (CDCl_3_, 150 MHz)

DEPT-135

DEPT-90

Figure S41. Simisyzygin H3 (10) DEPT spectrum

Figure S42. Simisyzygin H3 (10) HSQC spectrum

Figure S43. Simisyzygin H3 (10) COSY spectrum

Figure S44. Simisyzygin H3 (10) HMBC spectrum

Figure S45. Simisyzygin H3 (10) NOESY spectrum

Figure S46. Simisyzygin H4 (11) CD spectrum (MeOH)

Figure S47. Simisyzygin H4 (11) HRESIMS spectrum

Figure S48. Simisyzygin H4 (11) ^1^H NMR spectrum (CDCl_3_, 600 MHz)

Figure S49. Simisyzygin H4 (11) ^13^C NMR spectrum (CDCl_3_, 150 MHz)

DEPT-90

DEPT-135

Figure S50. Simisyzygin H4 (11) DEPT spectrum

Figure S51. Simisyzygin H4 (11) HSQC spectrum

Figure S52. Simisyzygin H4 (11) COSY spectrum

Figure S53. Simisyzygin H4 (11) HMBC spectrum

Figure S54. Simisyzygin H4 (11) NOESY spectrum

Figure S55. Simisyzygin I1 (12) CD spectrum (MeOH)

Figure S56. Simisyzygin I1 (12) HRESIMS spectrum

Figure S57. Simisyzygin I1 (12) ^1^H NMR spectrum (CDCl_3_, 400 MHz)

Figure S58. Simisyzygin I1 (12) ^13^C NMR spectrum (CDCl_3_, 100 MHz)

DEPT-90

DEPT-135

Figure S59. Simisyzygin I1 (12) DEPT spectrum

Figure S60. Simisyzygin I1 (12) HSQC spectrum

Figure S61. Simisyzygin I1 (12) COSY spectrum

Figure S62. Simisyzygin I1 (12) HMBC spectrum

Figure S63. Simisyzygin I1 (12) NOESY spectrum

Figure S64. Simisyzygin I2 (13) CD spectrum (MeOH)

Figure S65. Simisyzygin I2 (13) HRESIMS spectrum

Figure S66. Simisyzygin I2 (13) ^1^H NMR spectrum (CDCl_3_, 400 MHz)

Figure S67. Simisyzygin I2 (13) ^13^C NMR spectrum (CDCl_3_, 100 MHz)

DEPT-135

DEPT-90

Figure S68. Simisyzygin I2 (13) DEPT spectrum

Figure S69. Simisyzygin I2 (13) HSQC spectrum

Figure S70. Simisyzygin I2 (13) COSY spectrum

Figure S71. Simisyzygin I2 (13) HMBC spectrum

Figure S72. Simisyzygin I2 (13) NOESY spectrum

Figure S73. Simisyzygin I3 (14) CD spectrum (MeOH)

Figure S74. Simisyzygin I3 (14) HRESIMS spectrum

Figure S75. Simisyzygin I3 (14) ^1^H NMR spectrum (CDCl_3_, 600 MHz)

Figure S76. Simisyzygin I3 (14) ^13^C NMR spectrum (CDCl_3_, 150 MHz)

DEPT-135

DEPT-90

Figure S77. Simisyzygin I3 (14) DEPT spectrum

Figure S78. Simisyzygin I3 (14) HSQC spectrum

Figure S79. Simisyzygin I3 (14) COSY spectrum

Figure S80. Simisyzygin I3 (14) HMBC spectrum

Figure S81. Simisyzygin I3 (14) NOESY spectrum

Figure S82. Simisyzygin J1 (15) CD spectrum (MeOH)

Figure S83. Simisyzygin J1 (15) HRESIMS spectrum

Figure S84. Simisyzygin J1 (15) ^1^H NMR spectrum (CDCl_3_, 600 MHz)

Figure S85. Simisyzygin J1 (15) ^13^C NMR spectrum (CDCl_3_, 150 MHz)

DEPT-90

DEPT-135

Figure S86. Simisyzygin J1 (15) DEPT spectrum

Figure S87. Simisyzygin J1 (15) HSQC spectrum

Figure S88. Simisyzygin J1 (15) COSY spectrum

Figure S89. Simisyzygin J1 (15) HMBC spectrum

Figure S90. Simisyzygin J1 (15) NOESY spectrum

Figure S91. Simisyzygin J2 (16) CD spectrum (MeOH)

Figure S92. Simisyzygin J2 (16) HRESIMS spectrum

Figure S93. Simisyzygin J2 (16) ^1^H NMR spectrum (CDCl_3_, 600 MHz)

Figure S94. Simisyzygin J2 (16) ^13^C NMR spectrum (CDCl_3_, 150 MHz)

DEPT-135

DEPT-90

Figure S95. Simisyzygin J2 (16) DEPT spectrum

Figure S96. Simisyzygin J2 (16) HSQC spectrum

Figure S97. Simisyzygin J2 (16) COSY spectrum

Figure S98. Simisyzygin J2 (16) HMBC spectrum

Figure S99. Simisyzygin J2 (16) NOESY spectrum

Figure S100. Simisyzygin K1 (17) CD spectrum (MeOH)

Figure S101. Simisyzygin K1 (17) HRESIMS spectrum

Figure S102. Simisyzygin K1 (17) ^1^H NMR spectrum (CDCl_3_, 600 MHz)

Figure S103. Simisyzygin K1 (17) ^13^C NMR spectrum (CDCl_3_, 150 MHz)

DEPT-135

DEPT-90

Figure S104. Simisyzygin K1 (17) DEPT spectrum

Figure S105. Simisyzygin K1 (17) HSQC spectrum

Figure S106. Simisyzygin K1 (17) COSY spectrum

Figure S107. Simisyzygin K1 (17) HMBC spectrum

Figure S108. Simisyzygin K1 (17) NOESY spectrum

Figure S109. Simisyzygin K2 (18) CD spectrum (MeOH)

Figure S110. Simisyzygin K2 (18) HRESIMS spectrum

Figure S111. Simisyzygin K2 (18) ^1^H NMR spectrum (CDCl_3_, 600 MHz)

Figure S112. Simisyzygin K2 (18) ^13^C NMR spectrum (CDCl_3_, 150 MHz)

DEPT-135

DEPT-90

Figure S113. Simisyzygin K2 (18) DEPT spectrum

Figure S114. Simisyzygin K2 (18) HSQC spectrum

Figure S115. Simisyzygin K2 (18) COSY spectrum

Figure S116. Simisyzygin K2 (18) HMBC spectrum

Figure S117. Simisyzygin K2 (18) NOESY spectrum

Figure S118. Superoxide anion generation of different concentrations of compounds 1-8, 12, 16, 19 and LY294002

Figure S119. Elastase release of different concentrations of compounds 1-8, 12, 16, 19 and LY294002

**Analytical HPLC data** **for determination of purity of 3**

**Figure S120.** HPLC purity analysis of compound **3** (peak #3, 95.047% purity).

**X-ray diffraction data for 1**

The X-ray diffraction data of **1** were measured on a Bruker APEX DUO diffractometer equipped with an APEX II 4K CCD detector using Cu Kα radiation (*λ* = 1.54178 Å).

*Crystal data for simisyzygin A (****1****):* C_32_H_40_O_6_, *M* = 520.64, size 0.25 × 0.22 × 0.19 mm^3^, monoclinic, space group *P*2_1_, *a* = 12.6044(2) Å, *b* = 8.5586(2) Å, *c* = 12.8842(2) Å, α = *γ* = 90°, β = 96.9970(10)°, *V* = 1379.54(4) Å^3^, *T* = 100(2) K, *Z* = 2, *d*_calcd_ = 1.253 Mg/m^3^, *λ*(Cu Kα) = 1.54178 Å, *F*(000) = 560, reflections collected/independent reflections 26185/4384 [*R*(int) = 0.0223], *h* (−15/15), *k* (−10/7), *l* (−15/15), final *R* indices *R*_1_ = 0.0269 and *wR*_2_ = 0.0744, GOF on F^2^ = 1.142, absolute structure parameter = 0.11(4).

X-ray crystallographic analysis was performed at the Instrumentation Center of National Tsing Hua University. CCDC 2539079 contains the supplementary crystallographic data for compound **1**. These data can be obtained free of charge via www.ccdc.cam.ac.uk/data_request/cif, or by emailing data_request@ccdc.cam.ac.uk, or by contacting The Cambridge Crystallographic Data Centre, 12 Union Road, Cambridge CB2 1EZ, UK; fax: +44 1223 336033.
